# Novel *Hydrurus* species (Chrysophyceae) and their adaptations to high‐altitude European and Arctic snowfields

**DOI:** 10.1111/jpy.70162

**Published:** 2026-04-29

**Authors:** Lenka Procházková, Robert A. Andersen, Thomas Leya, Tomáš Řezanka, Martin Lukeš, Linda Nedbalová, Daniel Remias

**Affiliations:** ^1^ Department of Ecology, Faculty of Science Charles University Prague Czech Republic; ^2^ Friday Harbor Laboratories University of Washington Friday Harbor Washington USA; ^3^ Fraunhofer Institute for Cell Therapy and Immunology, Branch Bioanalytics and Bioprocesses IZI‐BB Potsdam Germany; ^4^ Institute of Microbiology, Czech Academy of Sciences Prague Czech Republic; ^5^ Laboratory of Algal Biotechnology Institute of Microbiology, Czech Academy of Sciences – Centre Algatech Třeboň Czech Republic; ^6^ Department of Environment & Biodiversity University of Salzburg Salzburg Austria

**Keywords:** fatty acids, fluorimetry, *Hydrurus*, phylogeny, pigments, polyols, snow algae

## Abstract

Colored snow caused by green algae (Chlorophyceae) is well known, but melting snowpacks can also harbor golden‐brown blooms consisting of Chrysophyceae. We collected 14 samples of cryoflora in the Austrian and Swiss Alps, the High Tatras in Slovakia, and in Arctic Svalbard. Eight laboratory unicellular flagellated strains were established from eight sites and phylogenetic analyses (18S rRNA and *rbc*L gene sequences) revealed new taxa belonging to the order Hydrurales (Chrysophyceae). Some formed tetrahedral swarmers; others were capsoid or amoeboid forms. Characteristics of vegetative cells and molecular markers, including the ITS2 rRNA region, supported the description of eight species: *Hydrurus novisii* sp. nov., *H. klavenessii* sp. nov., *H. tatrae* sp. nov., *H. pulcher* sp. nov., *H. pascheri* sp. nov., *H. svalbardensis* sp. nov., *H. nivalis* sp. nov. and *H. nemcovae* sp. nov. Pulse‐amplitude‐modulate (PAM) fluorometry indicated that the photosystem II of Arctic populations was adapted to high light conditions. Abundant polyunsaturated fatty acids supported cell survival at temperatures around 0°C, and the composition of these acids differed among species. The cells contained compatible solutes that could act as antifreeze agents. The main carotenoid fucoxanthin caused the overall golden‐brown pigmentation. The closest relatives of the new species were reported from snow and cold mountain streams and lakes, indicating that these Hydruralian microalgae prefer low temperatures and elevated irradiation. The large number of new species discovered during this limited sampling campaign suggests the underestimated diversity of phototrophic microbes in melting snow. Consequently, the genus *Hydrurus* shows a similar high relevance for snow algae blooms as *Chloromonas* does within the green algae.

**Abbreviations**
α: slope in the light‐limited section of the photosynthesis‐irradiance curve
CCCryo: Culture Collection of Cryophilic Algae
DIC: differential interference contrast
eETR: the relative electron transport rate
EM: electron microscopy
FA: fatty acid
FAME: fatty acid methyl ester
GTR: general time reversible
HPIC: high‐pressure‐ion‐chromatography
HPLC: high‐performance liquid chromatography
*I*_k_
: light saturation point
LM: light microscopy
MAA: mycosporine‐like amino acid
ML: maximum likelihood
MUFA: monounsaturated fatty acid
NA: numeric aperture
NCBI: National Center for Biotechnology Information
OHH: Osmund Holm‐Hansen's (medium)
PAM: pulse‐amplitude modulate fluorimetry
PAR: photosynthetically active radiation
PCR: polymerase chain reaction
PFD: photon flux density
P–I curve: photosynthesis–irradiance curve
PSII: photosystem II
PUFA: polyunsaturated fatty acid
SAFA: saturated fatty acid
SEM: scanning electron microscopy
TEM: transmission electron microscopy

## INTRODUCTION

Long‐lasting, melting snowpacks in polar and alpine regions provide habitats for specialized microalgae thriving in the water film between ice crystals. There, cells must cope with low temperatures, extreme irradiation conditions, and freeze–thaw cycles (Hoham & Remias, 2020). Cellular adaptations include robust morphological stages as part of the life cycle, abundant metabolites like secondary pigments with antioxidant activity, or protective proteins such as ice‐structuring proteins (Fiołka et al., 2020; Leya, 2013; Nakashima et al., 2021; Raymond & Remias, 2019). Striking green or red snow blooms are mainly caused by specialized microalgae from the Chlamydomonadales (Chlorophyceae). Nonetheless, freshwater Chrysophyceae also cause snow colorations, sometimes addressed as “yellow snow” (Remias et al., 2013, 2020) or, more recently, golden‐brown snow (Ono et al., 2021). Generally, the fact that Chrysophyceae are a significant part of the cryoflora has been overlooked, and consequently, they have been less investigated compared to chlorophyte and streptophyte algae. For example, in the classical compendium for snow algae by Kol (1968), the chrysophytes are mentioned only briefly. As golden‐brown snow blooms are observed frequently, it can be assumed that they have been under‐reported in the literature due to the chrysophyte cells disintegrating quickly after collection from the field, which has hindered detailed studies so far. The lack of cell walls during vegetative growth makes the flagellates very sensitive to changing osmotic conditions, yet silicified endogenous resting stages, called stomatocysts, were reported to be formed as part of their life cycles (Holen, 2014).

Even though golden‐brown snow blooms probably play an important ecological role in terms of phototrophic productivity in otherwise terrestrial polar and high altitude regions with low productivity, we still lack information about their biogeography, abundances in snowpacks, photosynthetic performance, overall biodiversity, and the ecophysiology strategies used to successfully and quickly develop ephemeral blooms each melting season.

Chrysophytes as a group are ubiquitous, occurring in temperate regions and elsewhere. Detailed information on their distribution is becoming increasingly available, and they range from cosmopolitan species to highly geographically restricted species (Kristiansen, 2001; Škaloud et al., 2020). Chrysophytes were often reported to occur in cold, oligotrophic waters (Fritsch, 1935; Round, 1981). They were the dominating phototrophic microbes in brine‐channels of melting polar sea ice (Stoecker et al., 1998). They occurred in slush layers of frozen alpine mountain lakes (Felip et al., 2002), and they appeared in snow melt streams (Klaveness, 2017). Environmental sequencing showed that Chrysophyceae are abundant in the cryosphere in many regions of the world such as Iceland (Bradley et al., 2023), Svalbard (Remias et al., 2023), the Cascade Mountains (Van Hees et al., 2023), the Coast Range in British Colombia in Canada (Yakimovich et al., 2020), and the European Alps (Krug et al., 2020; Yan et al., 2025). *Hydrurus* is one of the Chrysophyceaen algal genera frequently observed in this environment (Klaveness et al., 2011; Klaveness & Lindstrøm, 2011). Although most sequences belonging to this genus have come from natural samples, knowledge regarding their diversity and ecophysiology remains scarce. Only one species of this genus, *H. foetidus*, is known so far as a thallus bearing, macroscopic alga in cold rivers, where it releases an offensive smell like that of decomposing fish. The major source of chrysophyte volatile compounds was found to be derviatives of polyunsaturated fatty acids (PUFAs) whereas aromatics and terpenoids were found to be less important odor sources. Septic‐smelling sulfides, such as dimethyldisulphide and dimethyltrisulphide (produced, for example, during amino acid breakdown) have been found in cultures of *Chlorochromonas danica*, *Poterioochromonas malhamensis*, and *Synura petersenii* (Watson et al., 2001). Under conditions where *Hydrurus* *foetidus* is highly abundant in a stream, its characteristic odor can be detected (Lund & Lund, 1998 in Krizmanic et al., 2008). More recently, populations of unicellular species were observed in polar snow (Remias et al., 2013). It should be noted that there are other species within the Chrysophyceae that are not related to the Hydrurales that also cause golden‐brown snow: *Kremastochrysopsis austriaca* in the European Alps (Remias et al., 2020) and *Ochromonas*‐like sp. in Maritime Antarctica (Luo et al., 2020).

The aim of this study was to identify and characterize Chrysophycean algae isolated from snow blooms. We studied several High Arctic and Alpine sites in Europe, collected golden‐brown snow, and characterized their habitats. We measured rapid light curves in the native populations to reveal irradiation optima and established laboratory strains to study cell morphology and biochemistry (fatty acids, soluble carbohydrates, and carotenoid pigmentation).

## MATERIALS AND METHODS

### Sampling and cell counting

Field samples were collected in the Austrian Alps, the Swiss Alps, the High Tatras in Slovakia, and Svalbard during the snow melting seasons from 2018 to 2024 (Table 1, Figure S1). Golden‐brown blooms were observed either on the snow surface as faint spots or as thin layers (ranging from faint to dark) deeper within the snowpack. In the Alps, surface spots typically developed during very foggy (“bad”) weather, while deeper golden‐brown layers were found on sunny days. During foggy weather in mid‐June around noon, photosynthetically active radiation (PAR) reached 89–95 μmol photons · m^−2^ · s^−1^ (site WP395), whereas during clear skies it reached up to 2170 μmol photons · m^−2^ · s^−1^ (measured with a DeltaOhm HD 2302.0 LightMeter, cosine corrected sensors: LP471 UVA, UVB, and PAR; Italy). On Svalbard, algae occurred on the surface or in deep snow layers depending on the timing of sampling in the season and weather conditions prior to the harvest (e.g., recent snowfall from previous days resulted in algal blooms hiding below fresh snow) and the character of the locality. For surface‐apparent blooms, a thin layer of approximately 0.5 cm surface snow was removed with a sterile stainless steel scoop to reduce the content of dark snow detritus. For blooms in deeper snow layers, a sterile large shovel was used to remove centimeters to dozens of centimeters of white surface‐snow layers. The presence of chrysophyte cryoflora blooms was confirmed immediately with a field microscope (EM1 portable microscope). After these steps, samples were directly scooped up with freshly opened sterile 15‐ or 50‐mL centrifugation tubes, with care taken to ensure that only the inner surface of the tube contacted the snow sample. The tubes were then capped and placed in stainless steel thermos bottles to keep the samples cool until further processing. For samples WP225, WP264, WP271, and WP301, 2‐mL subsamples were fixed immediately by adding a drop of acidic Lugol's solution (10% dilute acetic acid). The determination of algal cell concentrations per snow meltwater followed the protocol described in Procházková et al. (2018). Values for electrical conductivity were obtained with WTW instruments (Weilheim, Germany, Cond 340i and Multi 9310).

**TABLE 1:** Sampling locations of snow in the Austrian and Swiss Alps, the High Tatras (Slovakia), and Arctic Svalbard (Norway), including sample codes, collection date, altitude (m a.s.l.) and geographic position (GPS) (AT, Austria; NO, Svalbard (Norway); SK, Slovakia; CH, Switzerland), and electrical conductivity (EC, in μS cm^−1^) of the meltwater.

Sample ID	Date	Location	Altitude (m a.s.l.)	GPS	EC
Sva10‐3^2^*	12. 07. 2010	NO (0), Svalbard	401	78.1868333N, 15.5645000E	3.8
CCCryo 533a‐19^1^	02. 06. 2017	CH (1), Uri Alps	2379	46.6016669N, 8.4652781E	n.d.
WP195^1^	17. 06. 2018	SK (2), High Tatras	2022	49.1756167N, 20.1570500E	n.d.
WP198^2,3^	03. 07. 2018	NO (3), Svalbard	161	78.1890500N, 15.5289333E	14
WP201^2,3^	04. 07. 2018	NO (4), Svalbard	302	78.2045500N, 15.6753333E	64
WP203^2,3,4^	05. 07. 2018	NO (5), Svalbard	525	78.2008833N, 15.4652167E	39
WP222.2^1^	16. 06. 2019	AT (6), High Tauern	2147	47.1268000N, 12.8142667E	2
WP225^1^	16. 06. 2019	AT (7), High Tauern	2112	47.1265000N, 12.8132000E	n.d.
WP227.2^1^	18. 06. 2019	AT (8), High Tauern	1933	47.1246833N, 12.8096500E	17
WP264^1^	13. 06. 2021	AT (9), Schladminger Tauern	1915	47.2412333N, 13.5143000E	7
WP271^1^	17. 06. 2021	AT (10), High Tauern	2132	47.1268500N, 12.8141167E	2
WP301^1,3,4,5^	17. 07. 2022	NO (11), Svalbard	565	78.2001500N, 15.4597833E	23
WP395^4,5^	11. 06. 2024	AT (12), High Tauern	1745	47.1335333N, 12.8078167E	4
WP401^4,5^	12. 06. 2024	AT (13), High Tauern	1813	47.1347500N, 12.8098833E	5
WP403^5^	12. 06. 2024	AT (14), High Tauern	1836	47.1310833N, 12.8074667E	n.d.

*Note*: The numbers in parentheses indicate the field sites in the map in Figure S1. Different purposes of collected samples shown by numbers: ^1^Indicate the snow samples out of which the newly described species were isolated. ^2^Sanger sequencing of field blooms. ^3^Rapid light curves of field populations. ^4^Fatty acids analysis of field blooms. ^5^Snow chemistry analysis. *Sample obtained during research of Remias et al. (2013).

### Strain isolation

Cells were cultivated in petri dishes with solidified DY‐V medium (1.4% agar w/v, recipe in Table S1). Unialgal colonies were picked from the agar plate, transferred to new agar plates, and kept at 5°C under diurnal cycle of 16:8 h light:dark (light: 20–50 μmol photons · m^−2^ · s^−1^). Backup plates on DY‐V agar were kept for 6 months prior to reinoculation at 5°C and approximately 20–40 μmol photons · m^−2^ · s^−1^. New plates were kept under the same conditions. In the case of WP222.2, liquid 20% DY‐V was used; in the case of CCCryo 533a‐19, liquid Osmund Holm‐Hansen's medium (OHH medium; Holm‐Hansen, 1964) modified by Sutton (1970) was used, and purification was achieved by serial dilution in multiwell‐plates.

### Microscopy

For electron microscopy (EM) and light (LM) microscopy, strains were grown in liquid medium at 5°C under diurnal cycle of 8:16 h light:dark (light: 80 μmol photons · m^−2^ · s^−1^) with constant gentle shaking in Erlenmeyer flasks (70 rpm). For transmission electron microscopy (TEM), cells were fixed as described in Procházková et al. (2018). Transmission electron microscopy grids were examined with a JEOL 1011 TEM (JEOL Ltd., Tokyo, Japan) at 80 kV. Photomicrographs were taken with a Veleta CCD camera and iTEM 5.1 software (Olympus Soft Imaging Solution GmbH, Münster, Germany). For scanning electron microscopy (SEM), strain cells were fixed on ice as described in Hanousková et al. (2019), and SEM gold‐coated coverslips were observed at 80 V with a JEOL 6380 LV (JEOL Ltd.). For confocal microscopy, strains WP301 and WP222.2 were examined with a Leica TCS SP2 with AOBS (Acousto‐Optical Beam Splitter), with the microscope stage cooled to 5°C. During LM, the chrysophytes changed their cell shapes quickly when exposed under cover slips. To avoid heat stress and cell deformations, the microscope slide had to be cooled prior to preparation and microscopic photos had to be taken quickly. Lugol's stain was used to show the second flagellum only. Light microscopy for strains WP264 and WP222.2 were performed on an Olympus BX43 at 1000× magnification using oil immersion, equipped with an Olympus DP27 camera (Olympus Europe SE, Hamburg, Germany). For strains WP225, WP227.2 and WP271, LM observations were made using a Leica DM RB light microscope (Leica Microsystems GmbH, Wetzlar, Germany) equipped with differential interference contrast (DIC), phase contrast, darkfield and brightfield optics and a Micro Tech LM Scope camera tube (Micro Tech Lab). Observations were made quickly using temporary wet mount microscope slides, with slides cooled on frozen ice packs between observations. Images were captured using a Canon 6D Mark II DSLR camera (Canon USA., Inc., Melville, New York, United States). For strain CCCryo 533a‐19, light microscopic observations were made at 400× magnification (40× lens Plan‐Neofluar with a numeric aperture, NA, of 0.75) and at 630× magnification (63× oil lens Plan Apochromat with an NA of 1.4) using a Zeiss Axioskop 2plus equipped with DIC optics (Carl Zeiss Microscopy Deutschland GmbH, Oberkochen, Germany) and a Colorview III camera (Olympus Soft Imaging Solution GmbH, Hamburg, Germany). The algae were observed and photographed within 5 min of sampling, after which time they tended to disintegrate on the microscope slide. For strains WP264, WP222.2, WP195, and WP301 light microscopic observations were made using a Nikon Eclipse 80i.

### Isolation of DNA, polymerase chain reaction sequencing

Total genomic DNA was extracted from the algal strains and field samples (if strains were not established) as described in Procházková et al. (2018). The 18S rRNA and *rbc*L genes were amplified by polymerase chain reaction (PCR) using the primers described in Remias et al. (2020). The internal transcribed spacer 2 (ITS2) rRNA region was amplified using the following primers: Kn3.1 (ACAACGATGAAGAACGCAGC; Wee et al., 2001) or TW81 (GGGATCCGTTTCCGTAGGTGAACCTGC; Goff et al., 1994) as the forward primer and AB28 (GGGATCCATATGCTTAAGTTCAGCGGGT; Goff et al., 1994) as the reverse primer. Amplification and sequencing reactions for these markers were identical to those described by Procházková et al. (2018). The newly generated sequences of the strains are available under GenBank accession numbers listed in Table 2.

**TABLE 2:** List of accession numbers of the *Hydrurus* strains (s) and field samples (f) obtained in this study.

Species	Code of field sample or strain	18S rRNA gene	ITS2 rRNA region	*rbc*L gene
*Hydrurus klavenessii*	WP222.2/CCCryo 572–25 (s)	PX339953	PX353697	
*Hydrurus nemcovae*	WP271/CCCryo 577–25 (s)	PX328972	PX352565	PX353345
*Hydrurus nivalis*	WP225/CCCryo 576–25 (s)	PX328971	PX352564	PX353346
*Hydrurus novisii*	WP264/CCCryo 571–25 (s)	PX339956	PX339957	PX353344
*Hydrurus pascheri*	WP227.2/CCCryo 574–25 (s)	PX328970	PX352563	
*Hydrurus pulcher*	CCCryo 533a‐19 (s)	PX339955	PX339958	
*Hydrurus* sp.	WP198 (f)	PX443430	PX519336	
*Hydrurus* sp.	WP201 (f)	PX443441	PX519337	
*Hydrurus* sp.	WP203 (f)	PX443445	PX519338	
*Hydrurus* sp.	Sva10‐3 (f)		PX519339	
*Hydrurus svalbardensis*	WP301/CCCryo 575–25 (s)	PX521730	PX519335	
*Hydrurus tatrae*	WP195/CCCryo 573–25 (s)	PX339954	PX353590	

### Molecular phylogeny

Two different alignments were constructed for the phylogenetic analyses, based on the 18S rRNA and *rbc*L gene sequences obtained from National Center for Biotechnology Information (NCBI) and in the course of this study. The sequences were selected according to the publications of Kristiansen and Škaloud (2017) and Andersen (2007), to encompass all Chrysophycean lineages. The 18S rRNA gene alignment contained 108 sequences (1687 bp); the initial *rbc*L gene matrix consisted of 52 sequences (933 bp). The outgroup taxa (*Synchroma* and *Nannochloropsis*) were selected according to the results of the recent multigene phylogenetic analysis of stramenopiles by Yang et al. (2012). The best‐fit nucleotide substitution model was estimated by jModeltest 2.0.1 (Posada, 2008). Based on the Akaike information criterion, the GTR + I + G model was selected for the 18S rRNA gene. Three partitions were set for *rbc*L gene sequences, and the GTR + I + G substitution model was applied for each of three codon positions. The 18S rRNA and *rbc*L gene phylogenetic trees were inferred by Bayesian inference and maximum likelihood (ML) according to Remias et al. (2020). Convergence of the two cold chains was checked by the average standard deviation of split frequencies (0.002921 and 0.000682) for the 18S rRNA gene and *rbc*L gene dataset, respectively. Bootstrap analyses and Bayesian posterior probabilities were performed as described by Remias et al. (2020).

### Rapid light curves

Prior to photosynthesis measurements, samples were slowly melted overnight and kept for about 24 h after collection in the dark at 4–5°C. In vivo chlorophyll fluorescence parameters were measured with a pulse‐amplitude modulated (PAM) fluorometer (PAM 2500, Heinz Walz GmbH, Germany) in an ice bath at approximately 2°C in a 0.4‐mL chamber. Field collected cells were exposed to photon flux densities (PFD) of 5, 9, 34, 67, 104, 201, 366, 622, 984, 1389, 1666, and 2018 μmol photons · m^−2^ · s^−1^ for 30 s each. The relative electron transport rates (rETRs), the apparent quantum yield for electron transport (α) and the light saturation point, *I*_k_, were obtained. Four independent replicates were measured. The data points were fit to the model assuming no photoinhibition (Webb et al., 1974). For further technical details, see Procházková et al. (2018).

### Pigment analysis

The cells were grown in 300‐mL glass tubes (Kniese‐Röhren) bubbled with sterile filtered air (0.2 μm) at 3°C in OHH medium (pH 5.5) on at 16:8 h light:dark cycle. Irradiation was provided by fluorescent tubes Cool White and Fluora (1:1) at 13–16 μmol photons · m^−2^ · s^−1^. Samples were harvested when the culture reached a cell concentration of around 5 × 10^6^ cells · mL^−1^. Biomass was harvested by centrifugation (4500 × *g*, 4°C, 8 min). Samples were stored at −80°C until freeze‐drying overnight (Christ Alpha 1–4, Martin Christ Gefriertrocknungsanlagen GmbH, Osterode am Harz, Germany). Samples were extracted in triplicate after grinding, addition of a spatula tip full of CaCO_3_ (X0774, AppliChem GmbH, Darmstadt, Germany), and extraction in N,N‐dimethyformamide (227056, Sigma‐Aldrich), then analyzed by high‐performance liquid chromatography (HPLC; Prominence, Shimadzu, Darmstadt, Germany). Pigments were separated on a Merck Chromolith Performance RP‐18e column (100 × 4.6 mm, Merck KgaA, Darmstadt, Germany) using the eluent A consisting of acetonitrile, 87% (34967, Fluka); H_2_O, 7.6%; methanol, 3.3% (article no. 83638.320, VWR); hexane, 1% (article no. 83629.320, VWR); and TRIS, 0.2 M at pH 8.0, 1% (article no. T1503, Sigma‐Aldrich) and eluent B consisting of methanol/hexane at a volume ratio of 5/1. Eluent A and B were used with the following profile: 0–6 min A = 100%, 6–13 min change of A to 0% and B to 100%, 13–18 min B 100%, and 18–22 min immediate change from B to A = 100% for equilibration and preparation for the next sample. Peak identification and pigment quantification were performed using the software LabSolutions (Shimadzu Deutschland GmbH, Duisburg, Germany).

### Soluble carbohydrate analysis

The presence of soluble polyols (sugar alcohols and alditols) was qualitatively evaluated by high‐pressure‐ion‐chromatography (HPIC) with a Dionex ICS3000 unit, a CarboPAc MA1‐column (250 × 4 mm) as stationary phase operated at 30°C, an isocratic mobile phase (200 mM NaOH, CO_2_‐free) at a 0.4 mL · min^−1^ pump rate, and a runtime of 35 min and an injection volume of 10 μL. The system was calibrated with glycerol, xylitol, meso‐erythritol, mannitol, arabitol, and sorbitol (Sigma‐Aldrich). Lyophilized cells on reweighted glass fiber filters were extracted with a mortar and pestle, with everything precooled to −80°C and the resultant powder suspended in 4 mL methyl tert‐butyl ether. Soluble carbohydrates were collected firstly by phase separation of the organic extract against 4 mL 0.5% formic acid and secondly by post‐extraction of the dried cell pellet with 2 mL 0.5% formic acid. Both aqueous extracts were pooled and prepared for injection by centrifugation at 17,000 × *g* for 10 min.

### Lipid extraction and fatty acid methyl esters analysis (FAMEs)

For samples WP203 and WP301, the extraction procedure was based on the method of Bligh and Dyer (1959), and elution was done from a Sep‐Pak Vac Silica cartridge 35 cc (Waters; 10 g normal‐phase silica) by chloroform (neutral lipids), acetone (glycolipids), and methanol (phospholipids) according to Saunders and Horrocks (1984). All classes of lipids were saponified overnight in 10% KOH in methanol at room temperature. The structures of fatty acid methyl esters (FAMEs) analysis were confirmed by comparison with gas chromatography/mass spectrometry retention times and fragmentation patterns with those of standard FAMEs (Supelco, Prague; Řezanka et al., 2008; Dembitsky et al., 1993). Procedures were described in more detail in Procházková et al. (2018). For samples WP395 and WP401, the FAME analysis was performed by direct esterification of 1 mg of freeze‐dried biomass followed by gas chromatography with flame ionization detection and comparison to FAME standards as described in Střížek et al. (2023).

### Snow meltwater chemistry

We targeted colored snow: Snow subsamples with golden‐brown coloration (Arctic: WP301; Alps: WP395, WP401, WP403) were slowly melted at room temperature and snow meltwater was filtered through Whatman GF/C glass fiber filters and then frozen before analysis. Chemical analyses (pH, electrical conductivity, Ca^2+^, Mg^2+^, Na^+^, K^+^, ${\mathrm{NH}}_{4}^{+}‐N$, ${\mathrm{NO}}_{2}^{-}‐N$, ${\mathrm{NO}}_{3}^{-}‐N$, ${\mathrm{PO}}_{4}^{3-}‐P$, dissolved organic carbon, inorganic carbon) of snow meltwater were performed in the laboratory at the Institute of Botany CAS in Třeboň.

## RESULTS

New Chrysophyceaen snow algae were isolated and established from the eight investigated sites: at six sites (WP222.2, WP225, WP227.2, WP264, WP271, WP301) blooms were dominated by the Chrysophycean species, one site (WP195) was dominated by *Chloromonas*, and one site (CCCryo 533a‐19) by *Sanguina*.

### *Hydrurus* Agardh (1824) emend. Procházková, Leya, Remias & R.A. Andersen

Emended description: Either macroscopic, brown, sessile thalli with distinct apical growth or unicellular, free‐living cells. Cells oval to ovoid to dorsiventrally flattened, in thalli mostly clustered peripherally in the gelatinous substance. Free‐living cells flagellate, capsal, or ameboid. Cells with a parietal bilobed yellow‐brown chloroplast, with or without eyespot and/or a pyrenoid, where known; one or more pulsating vacuoles, and small shiny drops or granular spheres of chrysolaminarin in vacuoles present. Tetrahedral flagellates present or absent. Silica cysts (stomatocysts), where known, oval‐spherical with a semi‐ring‐shaped thickening.

Type species: *Hydrurus foetidus* (Villars) Trevisan.

### Taxonomic descriptions

**FIGURE 1:**
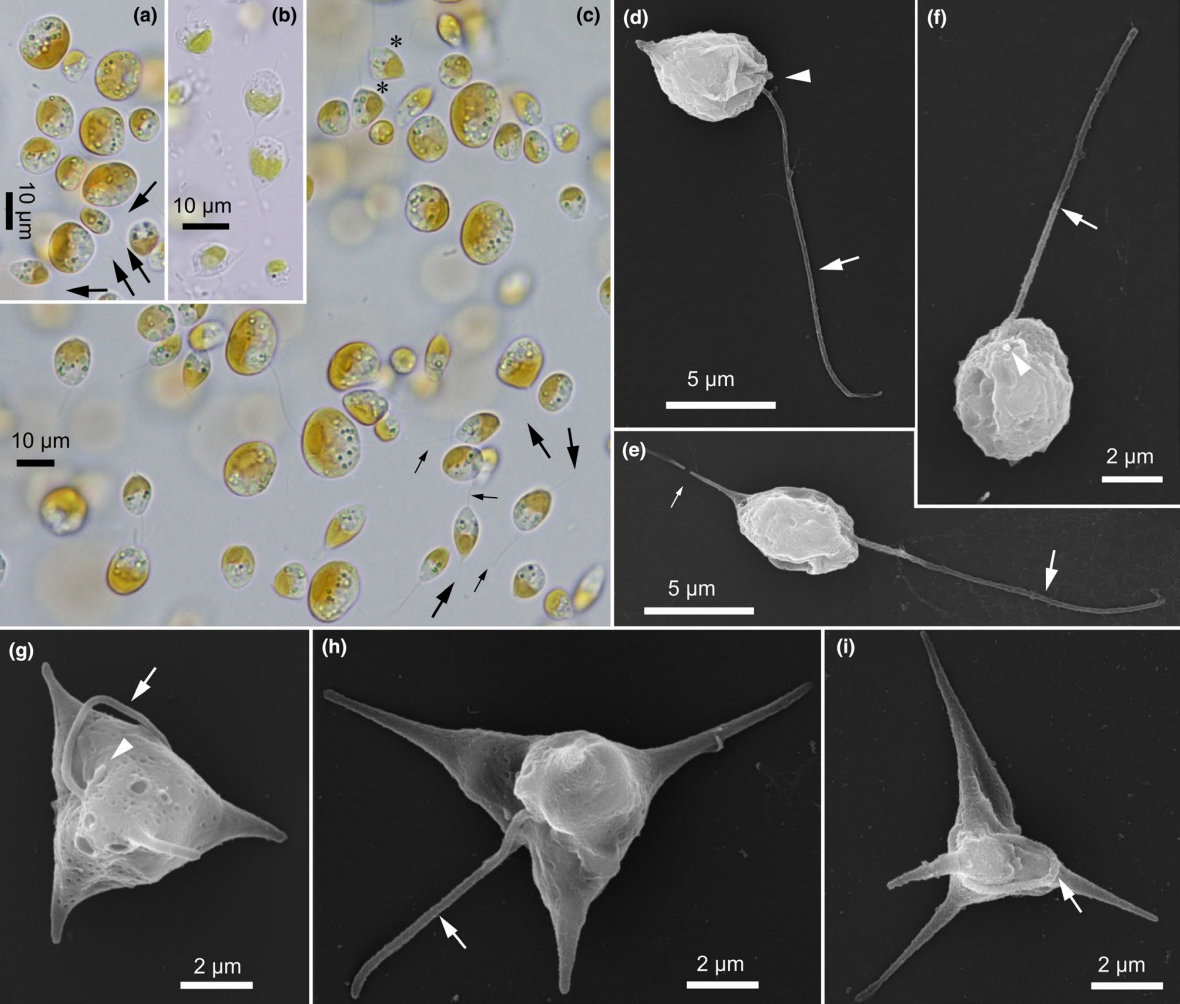
*Hydrurus novisii* sp. nov. WP264 (= CCCryo 571–25). LM (a–c), and SEM (d–i) images. (a and b) flagellated cells with one parietal chloroplast, large arrow indicates the long flagellum. (c) tetrahedral flagellates (asterisks) and flagellates with cytoplasmatic pseudofilament (little arrow) and long flagellum (large arrow). (d–f) flagellates with long flagellum (large arrow), very short flagellum (arrowhead) and cytoplasmatic pseudofilament (little arrow). (g–i) tetrahedral flagellates with long flagellum (large arrow) and very short flagellum (arrowhead).

***Hydrurus novisii* Procházková & Remias sp. nov. (Figure** **1** **)**

*Description:* unicellular, cells broadly ovoid, 4.9–13.4 μm wide and 8.1–14.3 μm long, one parietal chloroplast, without eyespot; one contractile vacuole; flagellated cells with one visible flagellum; however, a second very short flagellum can be observed by EM; healthy cells with numerous lipid droplets and a large chrysolaminarin droplet; tetrahedral flagellates are usually present; capsoid and amoeboid cells were observed; at higher light intensities (100 μmol photons · m^−2^ · s^−1^), both flagellates and immotile cell stages were observed; macroscopic pseudothalli may occur; stomatocysts unknown. In dividing cells, the chloroplast cleaves before the nucleus. Cytokinesis involves cell elongation, as has been observed in most chrysophytes. ITS2 rRNA region sequence unique.

*Holotype here designated:* Portion of a single gathering of cells on SEM stub deposited in at the Culture Collection of Algae of Charles University in Prague (http://botany.natur.cuni.cz/algo/caup.html) as the item CAUP‐TYPE‐50, material consists of gold‐coated flagellates and tetrahedral flagellates from the authentic culture strain CCCryo 571‐25 in a metabolically inactive state (GenBank accession no. PX339957 ITS2; culture no. WP264/CCCryo 571–25, derived from field collection) 13 June 2021, a golden‐brown stripe about 1 cm below snow surface in a small snow field close to alpine shrubs along a hiking path from Felseralm to Wildsee via Bödenalm, near Obertauern, Schladminger Tauern, Austria, 47°14.474′ N, 13°30.858′ E, 1915 m a.s.l.

*Authentic culture strain*: WP264. The strain was deposited at the CCCryo Culture Collection of Cryophilic Algae (CCCryo, available online: http://cccryo.fraunhofer.de/web/strains) in Potsdam, Germany, as a living culture as strain number CCCryo 571‐25.

*Etymology:* The species epithet “*novisii*” is in honor of Dr. Phil Novis, Landcare Research, New Zealand, who has made significant contributions to the ecology and physiology of microalgae inhabiting snow, ponds and soil (Novis, 2002a, 2002b; Novis et al., 2024, 2025).

**FIGURE 2:**
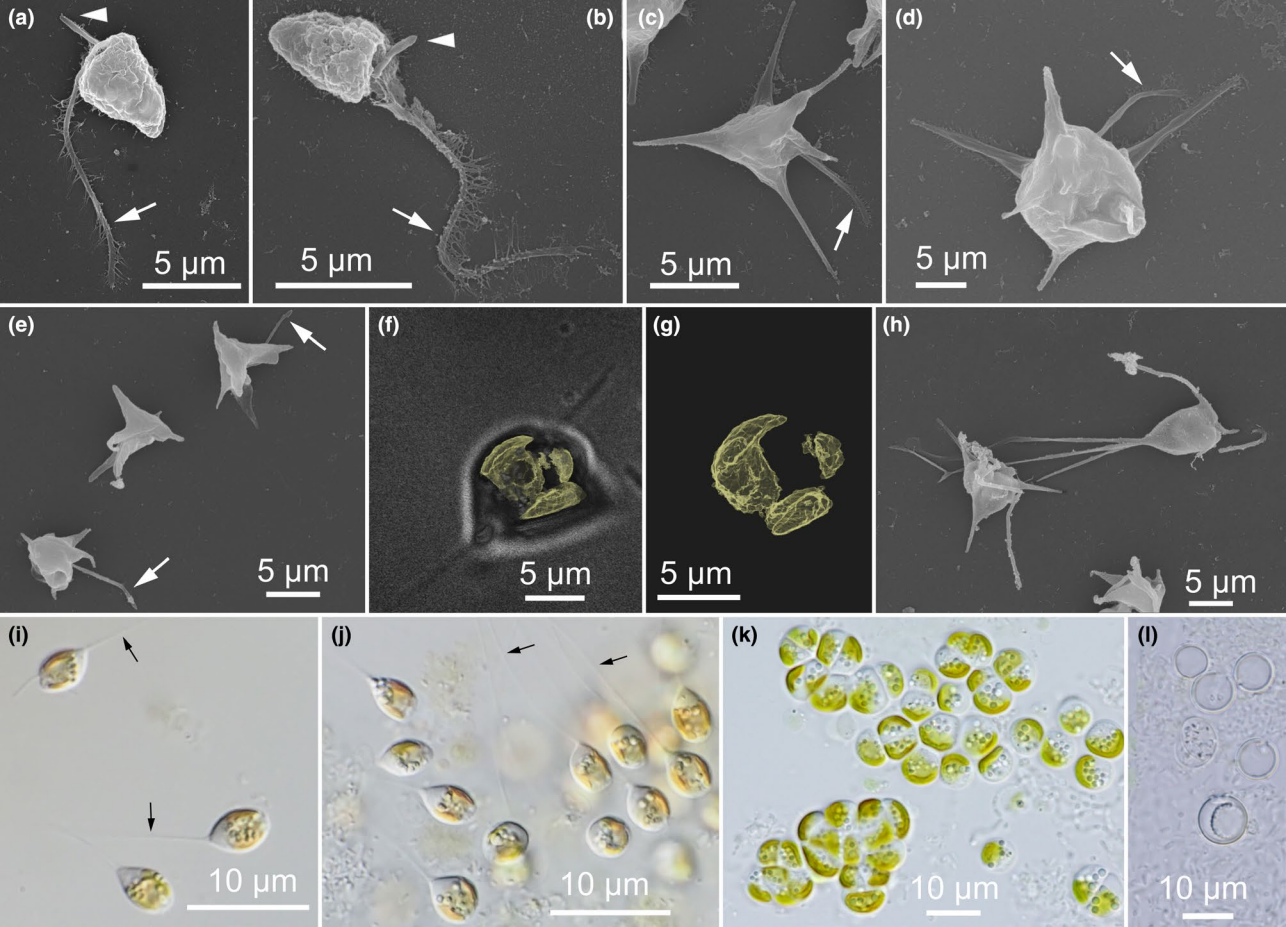
*Hydrurus klavenessii* sp. nov. WP222.2 (= CCCryo 572–25). SEM (a–e, h), confocal (f, g) and LM (i–l) images. (a, b) The long flagellum (large arrow) and the short flagellum (arrowhead), (c–g) tetrahedral to heptahedral flagellates with detailed structure of chloroplasts shown in (f and g) and long flagellum (large arrow). (h–j) cell with several pseudofilaments or rhizopodia extending from plasmalemma (small arrow), parietal chloroplast and lipid bodies, (k) capsal stage, (l) empty, smooth‐walled stomatocysts. Scale bar indicated.

***Hydrurus klavenessii* Procházková & Remias sp. nov. (Figure** **2** **)**

*Description:* unicellular, cells ovoid 4.1–8.3 μm wide and 6.2–12.5 μm long, one to two parietal chloroplasts, without eyespot; one contractile vacuole; flagellated cells with one flagellum visible by LM; however, the second shorter flagellum is observable by EM; healthy cells with numerous lipid droplets and a large chrysolaminarin droplet; tetrahedral flagellates are dominating; capsoid and amoeboid cells were observed; stomatocysts unknown. In dividing cells, the chloroplast divides before the nucleus. Cytokinesis involves cell elongation like most typical chrysophytes. ITS2 rRNA region sequence unique.

*Holotype here designated*: Portion of a single gathering of cells on SEM stub, deposited at the Culture Collection of Algae of Charles University in Prague (http://botany.natur.cuni.cz/algo/caup.html) as the item CAUP‐TYPE‐51, material consists of gold coated predominated tetrahedral flagellates from the authentic culture strain CCCryo 572‐25 in a metabolically inactive state (GenBank accession no. PX353697 ITS2; culture no. WP222.2/CCCryo 572–25, derived from field collection) 16 June 2019, surface golden‐brown snow field near Großglockner Hochalpenstraße, Hohe Tauern, Austria, 47°07.608′ N 12°48.856′ E, 2147 m a.s.l.

*Authentic culture strain*: WP222.2. The strain was deposited at the CCCryo Culture Collection of Cryophilic Algae (CCCryo, available online: http://cccryo.fraunhofer.de/web/strains) in Potsdam, Germany, as a living culture, strain number CCCryo 572‐25.

*Etymology:* The species epithet “*klavenessii*” is in memoriam Prof. Dag Klaveness (1945–2020), Norwegian limnologist who mainly worked with eukaryotic microorganisms in an ecological and evolutionary context. He was the first person to successfully cultivate *Hydrurus foetidus* in laboratory conditions (Klaveness, 2017; Klaveness & Lindstrøm, 2011).

**FIGURE 3:**
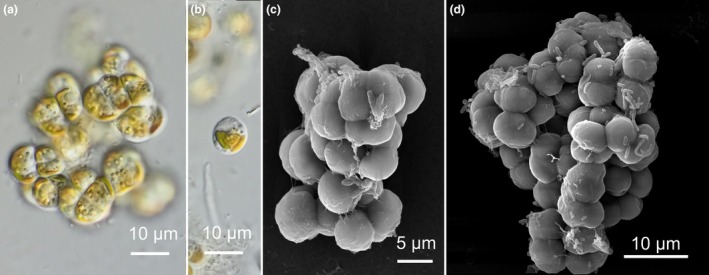
*Hydrurus tatrae* sp. nov. WP195 (= CCCryo 573‐25). LM (a, b), SEM (c, d). Capsal stages (a, c, d) and flagellated cell (b).

***Hydrurus tatrae* Procházková & Remias sp. nov. (Figure** **3** **)**

*Description:* unicellular, cells ovoid 3.4–9.4 μm wide and 5.0–9.8 μm long, one parietal chloroplast, without eyespot; one contractile vacuole; predominantly present on agar as capsal stages; tetrahedral flagellates noticed; other flagellates with one flagellum visible in LM rarely present, stomatocysts never observed. ITS2 rRNA region sequence unique.

*Holotype here designated:* Portion of a single gathering of cells on SEM stub, deposited at the Culture Collection of Algae of Charles University in Prague (http://botany.natur.cuni.cz/algo/caup.html) as the item CAUP‐TYPE‐52, material consists of gold‐coated capsal stages from the authentic culture strain CCCryo 573–25 in a metabolically inactive state (GenBank accession no. PX353590 ITS2; culture no. WP195/CCCryo 573‐25, derived from field collection) 17 June 2018, snowfield, scattered cells in a bloom of *Chloromonas* spp., Kotlina pod Prielomom, High Tatra Mountains, Slovakia. 49°10.537′ N, 20°09.423′ E, 2022 m a.s.l.

*Authentic culture strain*: WP195. The strain was deposited at the CCCryo Culture Collection of Cryophilic Algae (CCCryo, available online: http://cccryo.fraunhofer.de/web/strains) in Potsdam, Germany, as a living culture, strain number CCCryo 573‐25.

*Etymology:* The species epithet “tatrae” is based on the name of the geographic region, the Tatra Mountains, where it was found.

**FIGURE 4:**
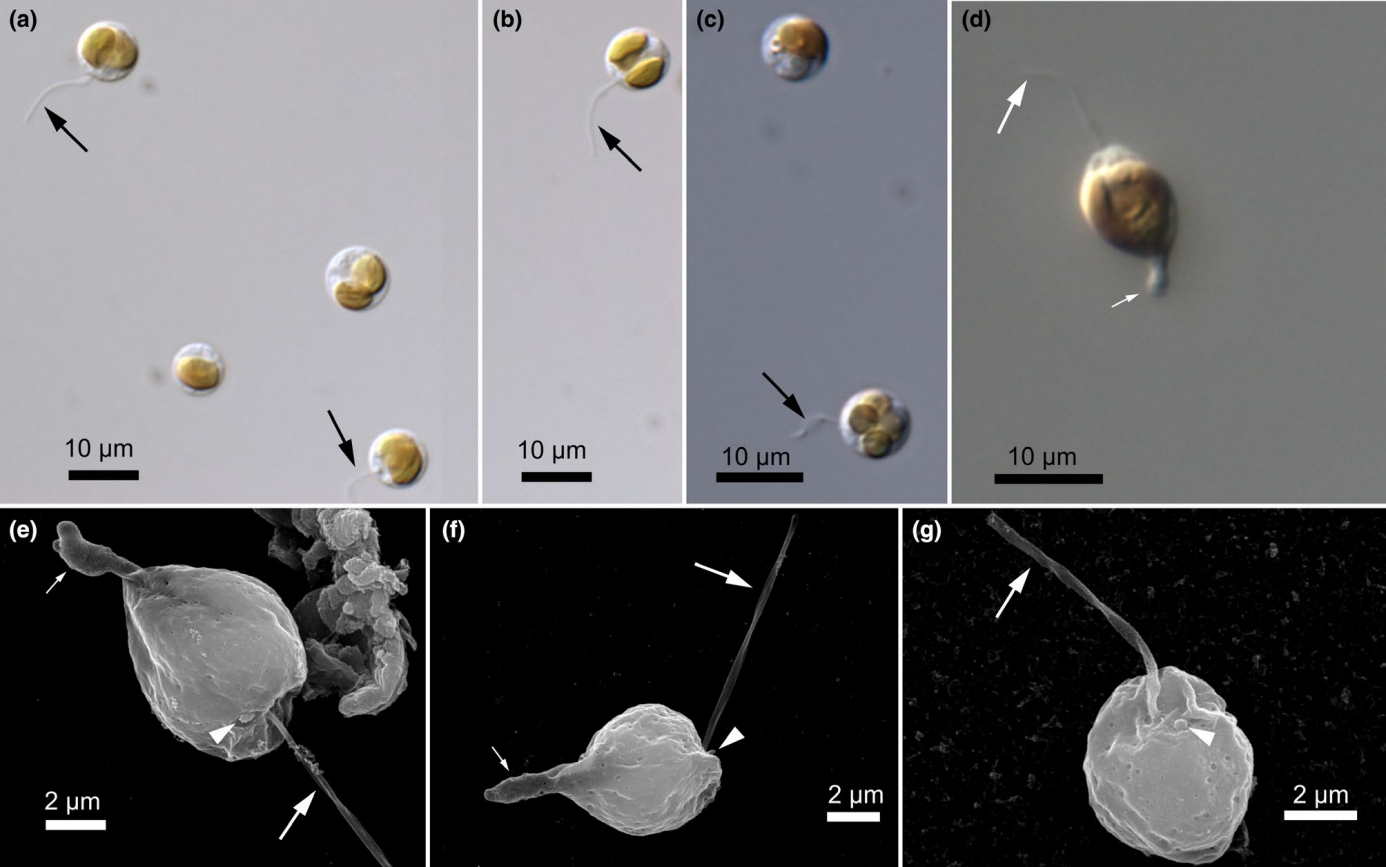
*Hydrurus pulcher* sp. nov. CCCryo 533a‐19. LM (a–d), SEM (e–g) images. (a and b) the long flagellum (large arrow) to be seen in living cells, one or two chloroplasts per cell, (c) transiently four chloroplasts per cell prior to cell division, (d) vegetative flagellate with the long flagellum (large arrow) on the cell apex and cytoplasmic projection (little arrow) on the cell antapex, (e–g) very short flagellum (arrowhead), long flagellum (large arrow) and cytoplasmic projection (little arrow).

***Hydrurus pulcher* Leya & Procházková sp. nov. (Figure** **4** **)**

*Description:* unicellular, motile cells ovoid 4.4–9.0 μm width and 5.9–9.4 μm length, with or without cytoplasmic projection on antapex, one to two parietal chloroplasts, without eyespot; one contractile vacuole; motile flagellate with one visible flagellum; however, a second very short flagellum can be observed by EM; tetrahedral flagellates are present; capsal stages observed, stomatocysts unknown. ITS2 rRNA region sequence unique.

*Holotype here designated:* Portion of a single gathering of cells on SEM stub, deposited at the Culture Collection of Algae of Charles University in Prague (http://botany.natur.cuni.cz/algo/caup.html) as the item CAUP‐TYPE*‐*53, material consists of gold‐coated flagellates from the authentic culture strain CCCryo 533a‐19 in a metabolically inactive state (GenBank accession no. PX339958 ITS2; culture no. CCCryo 533a‐19, derived from field collection) 02 June 2017, snowfield, scattered cells in a bloom of *Sanguina nivaloides*, Tiefenbach, municipality of Realp, Urseren, canton of Uri, Switzerland, 46.601667° N, 8.465278° E, 2379 m a.s.l.

*Authentic culture strain*: The strain was deposited at the CCCryo Culture Collection of Cryophilic Algae (CCCryo, available online: http://cccryo.fraunhofer.de/web/strains) in Potsdam, Germany, as a living culture as strain number CCCryo 533a‐19.

*Etymology:* The species epithet “*pulcher*” was chosen as snow fields with this species present are strikingly beautiful.

**FIGURE 5:**
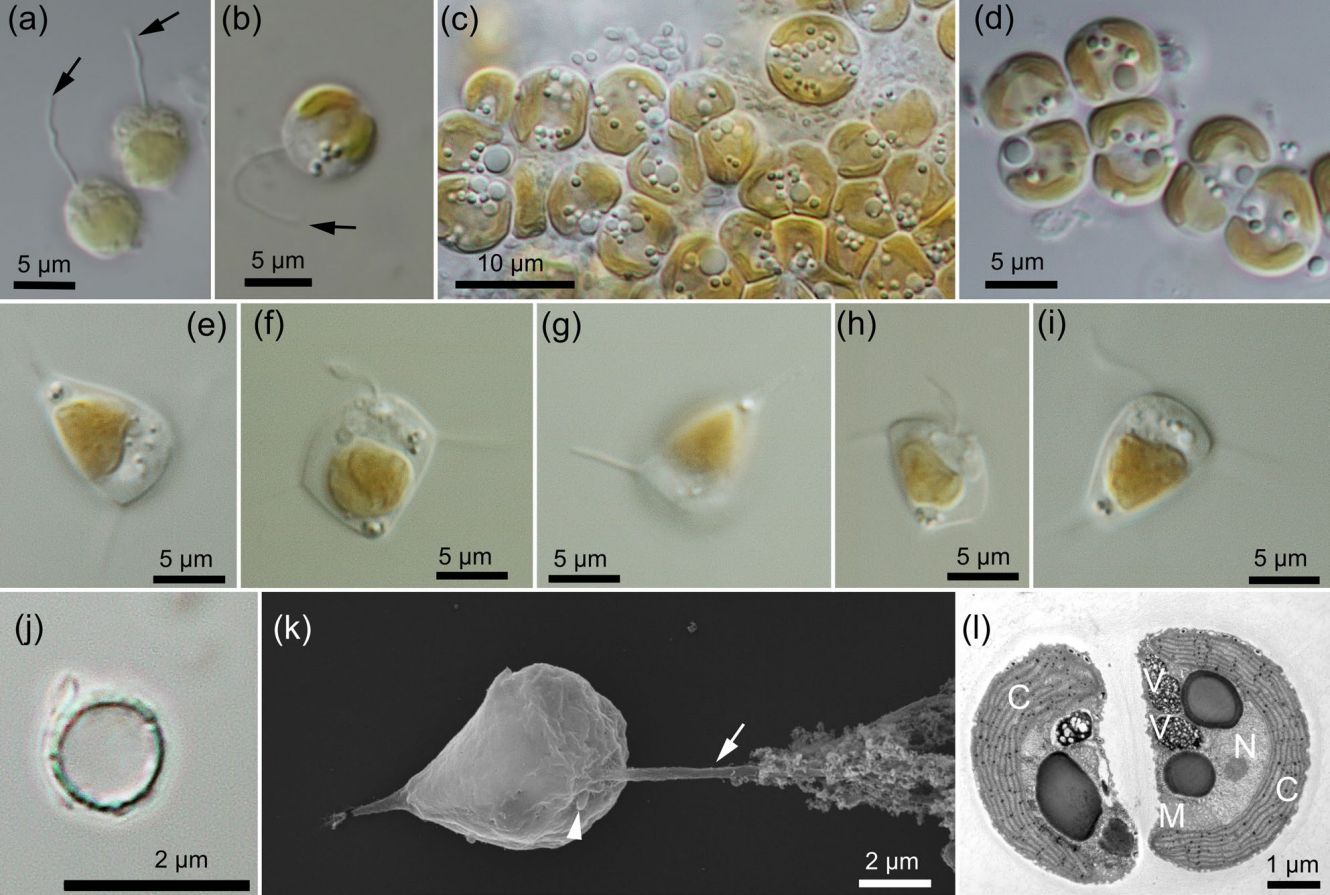
*Hydrurus pascheri* sp. nov. WP227.2 (= CCCryo 574‐25). LM (a–j), SEM (k), TEM (l) images. (a) the long flagellum (large arrow) visible in Lugol‐fixed subsample, (b) living flagellate, (c, d) non‐motile cells showing the parietal chloroplast and lipid droplets, (e–i) tetrahedral flagellates, (j) stomatocyst, (k) long (large arrow) and very short flagellum (arrowhead), (l) section of non‐motile cells showing parts of chloroplasts (C), mitochondria (M), nucleus (N), and the granular chrysolaminarin vacuoles (V).

***Hydrurus pascheri* Procházková, Remias & R.A. Andersen sp. nov. (Figure** **5** **)**

*Description:* unicellular, cells ovoid 4–7.8 μm wide and 4.8–8.5 μm long, one parietal chloroplast, without eyespot; one contractile vacuole; flagellates with one visible flagellum, a second very short flagellum visible by EM; tetrahedral flagellates present; capsal stages observed; stomatocysts unknown. ITS2 rRNA region sequence unique.

*Holotype here designated:* Portion of a single gathering of cells on TEM block, deposited at the Culture Collection of Algae of Charles University in Prague (http://botany.natur.cuni.cz/algo/caup.html), as the item CAUP‐TYPE‐54, material consists of resin‐embedded vegetative cells from the authentic culture strain CCCryo 574–25 in a metabolically inactive state (GenBank accession no. PX352563 ITS2; culture no. WP227.2/CCCryo 574‐25, derived from field collection) 18 June 2019, snowfield near mountain road, a golden‐brown stripe a few cm below snow surface, Fusch an der Glocknerstraße, Hohe Tauern, Austria. 47°07.481′ N, 12°48.579′ E, 1933 m a.s.l.

*Authentic culture strain*: WP227.2. The strain was deposited at the CCCryo Culture Collection of Cryophilic Algae (CCCryo, available online: http://cccryo.fraunhofer.de/web/strains) in Potsdam, Germany, as a living culture as strain number CCCryo 574‐25.

*Etymology:* The species epithet “*pascheri*” is in memoriam Adolf Pascher (1881–1945), professor at the German University in Prague, a prominent botanist, phycologist and limnologist notable for publishing more than 150 papers about protists, multicellular algae and vascular plants, he established the foundational concept of organizational levels in algal morphology (Mollenhauer, 2001).

**FIGURE 6:**
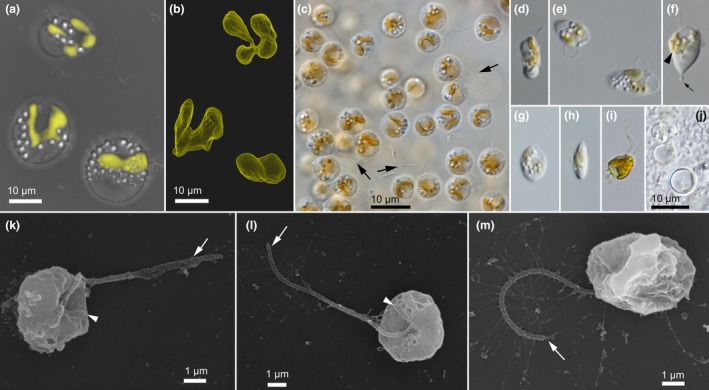
*Hydrurus svalbardensis* sp. nov. WP301 (= CCCryo 575‐25). LM (a, c–j), confocal laser scanning microscopy (b), SEM (k–m). (a) black‐white bright field picture showing cells with parietal (additionally colored) chloroplast and lipid droplets, (b) a single picture from the composite video assembled of the strain WP301, showing the connections retained within lobe of the chloroplast, (c) living field sample used for establishing the strain, long flagellum (large arrow), (d–h, j) strain cells showing (d) native elongated shape of cell from one side and (e) more ovoid shape cell from other side, (f) stigma‐bearing chloroplast (arrowhead) and cytoplasmic cell projection (little arrow), (g and h) ventrally flattened cell, (i) the long flagellum to be seen in Lugol‐fixed subsample, (j) stomatocyst, (k, l) long (large arrow) and very short flagellum (arrowhead), (m) long flagellum with mastigonemes (large arrow).

***Hydrurus svalbardensis* Remias & Procházková sp. nov. (Figure** **6** **)**

*Description:* elongated, dorsoventrally flattened unicell swarmers 3.4–7.8 μm wide and 6.9–11.7 μm long, one parietal chloroplast, with linear eyespot; one contractile vacuole; living flagellate cells with one visible flagellum, a second very short flagellum is visible by EM; tetrahedral flagellates not present; stomatocysts unknown. ITS2 rRNA region sequence unique.

*Holotype here designated:* Portion of a single gathering of cells on SEM stub, deposited at the Culture Collection of Algae of Charles University in Prague (http://botany.natur.cuni.cz/algo/caup.html) as the item CAUP‐TYPE‐55, material consists of gold‐coated vegetative cells from the authentic culture strain CCCryo 575‐25 in a metabolically inactive state (GenBank accession no. PX519335 ITS2; culture no. WP301/CCCryo 575‐25, derived from field collection) June 17, 2022, a dark, golden‐brown (slushy) snow below 5 cm of white snow on ca. 30° steep permanent ice plate north of Nordenskiöldfjellet peak and maybe formerly part of Platåbreen glacier, Svalbard, Norway. 78°12.009′ N, 15°27.587′ E, 565 m a.s.l., much fine (rock) debris present.

*Authentic culture strain*: WP301. The strain was deposited at the CCCryo Culture Collection of Cryophilic Algae (CCCryo, available online: http://cccryo.fraunhofer.de/web/strains) in Potsdam, Germany, as a living culture as strain number CCCryo 575‐25.

*Etymology:* The species epithet “*svalbardensis*” is based on the name of the geographic region where it was found, Svalbard, the Norwegian high Arctic Archipelago.

**FIGURE 7:**
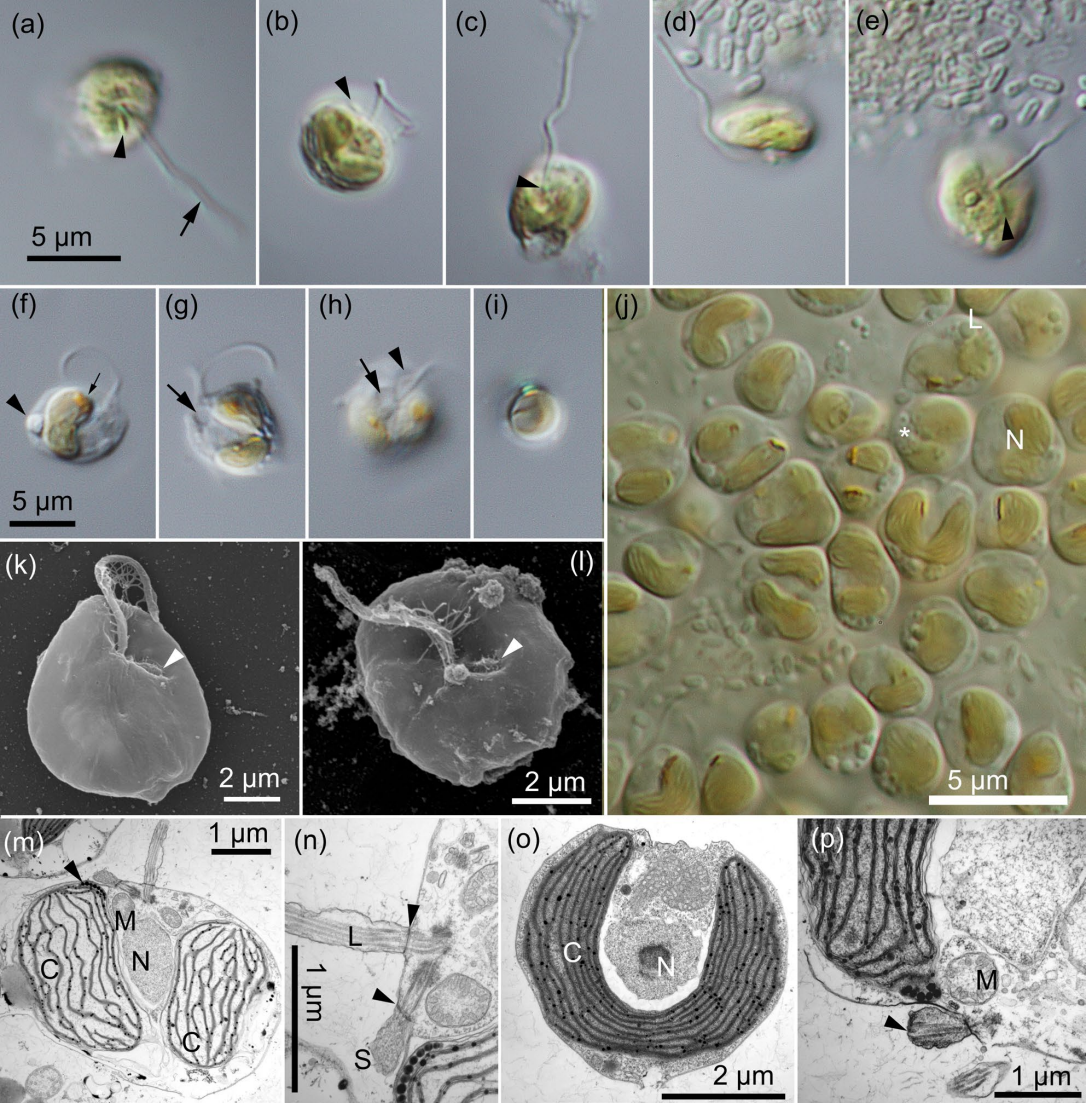
*Hydrurus nivalis* sp. nov. WP225 (= CCCryo 576–25). LM (a–j), SEM (k, l), TEM (m–p) images. (a–e) the long flagellum (large arrow) and short flagellum (arrowhead) to be seen in Lugol‐fixed subsample, (f) storage product (arrowhead) and red eyespot (little arrow), (g) bilobed chloroplast and contractile vacuole (large arrow), (h) contractile vacuole (large arrow) and probable R1 root (arrowhead), (i) stomatocyst with a collar, (j) chrysolaminaran vacuoles (asterisk), nucleus (N) and lipid droplet (L), (k, l) long and a very short flagellum (arrowhead), (m) section of cell showing two parts of chloroplast (C), mitochondria (M) and nucleus (N) and stigma (arrowhead), (n) longitudinal section of axonema and basal bodies (arrowhead) of long flagellum (L), short flagellum (S), and their basal bodies. Note the dense transitional plates (arrowheads), (o) parietal chloroplast (C) with central nucleus (N), (p) mitochondria (M) close to basal bodies of short flagellum (arrowhead).

***Hydrurus nivalis* R.A. Andersen, Remias & Procházková sp. nov. (Figure** **7** **)**

*Description:* unicellular, cells ovoid dorsiventrally flattened 3.1–6.5 μm wide and 4.1–8.2 μm long, flattened along the anterior–posterior axis; one parietal chloroplast with a prominent ellipsoid red eyespot; one contractile vacuole; living flagellate cells with one visible flagellum; however, a second very short flagellum is visible in Lugol's fixed cells and under electron microscopy; healthy cells with numerous lipid droplets and a large chrysolaminarin droplet; flagellate stage is predominant in liquid cultures; capsoid and amoeboid cells were observed; stomatocysts unknown; tetrahedral flagellates never observed. For dividing cells, the chloroplast divides before the nucleus. Cytokinesis involves cell elongation, as is typical for most chrysophytes. ITS2 rRNA region sequence unique.

*Designated holotype*: portion of a single gathering of cells on TEM block, deposited at the Culture Collection of Algae of Charles University in Prague (http://botany.natur.cuni.cz/algo/caup.html) as the item CAUP‐TYPE‐56, material consists of resin‐embedded vegetative cells from the culture strain CCCryo 576‐25 in a metabolically inactive state (GenBank accession no. PX352564 ITS2; culture no. WP225/CCCryo 576‐25, derived from field collection) 16 June 2019, flat, golden‐brown snowfield near the mountain road Großglockner Hochalpenstraße, Salzburg, Hohe Tauern, Austria, 47°07.590′ N, 12°48.792′ E, 2112 m a.s.l.

*Authentic culture strain*: WP225. The strain was deposited at the CCCryo Culture Collection of Cryophilic Algae (CCCryo, available online: http://cccryo.fraunhofer.de/web/strains) in Potsdam, Germany, as a living culture as strain number CCCryo 576‐25.

*Etymology:* the epithet “*nivalis*” refers to the snowy habitat where the alga was isolated from.

**FIGURE 8:**
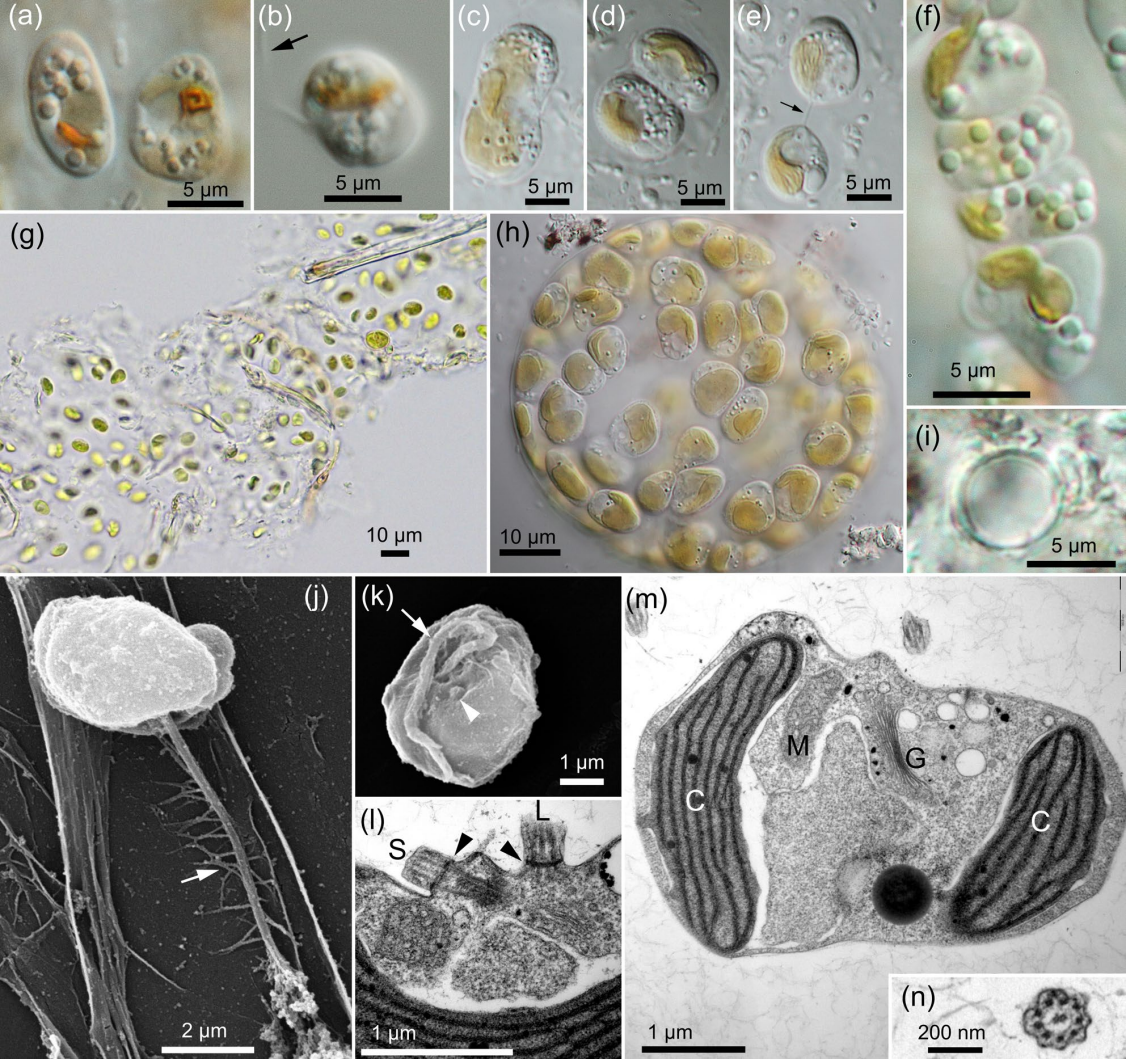
*Hydrurus nemcovae* sp. nov. WP271 (= CCCryo 577–25). LM (a–i), SEM (j, k), TEM (l–n) images. (a) Cell with longitudinal eyespot, (b) a flagellate with long flagellum (large arrow), (c–e) three different cell division stages, note cytoplasmatic strand connecting two daughter cells (little arrow), (f) linear division, (g) loosely developed thallus in liquid medium, (h) colony of about 128 cells, (i) stomatocyst, (j) cell with axoneme (large arrow), (k) cell with the long (large arrow) and short (arrowhead) flagellum, (l) longitudinal section of axoneme and basal bodies (arrowhead) of long flagellum (L), short flagellum (S), (m) cell section showing Golgi apparat (G), mitochondrion (M) and chloroplast section (C), (n) transverse section through the 9 + 2 axoneme of the long flagellum.

***Hydrurus nemcovae* Remias, R.A. Andersen & Procházková (Figure** **8** **)**

*Description:* unicellular, cells ovoid flattened along the anterior–posterior axis, 4.4–10.1 μm wide and 6.6–13.7 μm long; one parietal chloroplast with a prominent red eyespot; one contractile vacuole; flagellates with one visible flagellum visible in LM, the second short flagellum is visible using EM; vital cells with numerous lipid droplets and a large chrysolaminarin droplet; flagellated stages are predominant in liquid medium; tendency to form loosely developed “thalli” (individual cells arranged peripherally in joint mucilage) or huge aggregates (>120 cells), capsoid and amoeboid cells observed; stomatocysts unknown; tetrahedral flagellates never observed. ITS2 rRNA region sequence unique.

*Holotype here designated:* portion of a single gathering of cells on TEM block, deposited at the Culture Collection of Algae of the Charles University in Prague (http://botany.natur.cuni.cz/algo/caup.html) as the item CAUP‐TYPE‐57, material consists of resin‐embedded vegetative cells from the authentic culture strain CCCryo 577‐25 in metabolically inactive state (GenBank accession no. PX352565 ITS2; culture no. WP271/CCCryo 577‐25, derived from field collection) 17 June 2021, golden‐brown bloom 25 cm below snow surface in a snow field with a basal ice plate, near Großglockner Hochalpenstraße, Salzburg, Hohe Tauern, Austria. 47°07.611′ N 12°48.847′ E, 2132 m a.s.l.

*Authentic culture strain*: WP271. The strain was deposited at the CCCryo Culture Collection of Cryophilic Algae (CCCryo, available online: http://cccryo.fraunhofer.de/web/strains) in Potsdam, Germany, as a living culture as strain number CCCryo 577‐25.

*Etymology:* The species epithet “*nemcovae*” is in honor of Assoc. Prof. Yvonne Němcová, Prague, Czechia, who has contributed greatly to the taxonomy, physiology and pattern of distribution of chrysophytes (Nemcova et al., 2023, 2024; Nemcova & Rott, 2018).

### Morphological analyses

To investigate morphological differences among the eight new, closely related *Hydrurus* species in detail, we characterized each species by measuring the length and width of 30 randomly chosen cells. The species were obviously heterogeneous in the sizes of their cells (Figure S2). *Hydrurus svalbardensi*s, *H. nivalis*, and *H. nemcovae* differed from the other five *Hydrurus* species isolated from snow in the presence of stigma and in their dorsoventrally flattened cell shapes. In contrast, *H. novisii*, *H. klavenessii*, *H. tatrae*, *H. pulcher*, and *H*. *pascheri* developed ovoid flagellates and tetrahedral flagellates, but they never had stigma. A prominent ultrastructural feature (observable under SEM) of *H. klavenessii* was a 3 μm (length) short flagellum, whereas all other *Hydruru*s species had much shorter ones (0.5–1 μm long). The studied *Hydrurus* species had one long flagellum visible using light microscopy and a short one rarely to be seen and only in Lugol‐fixed samples using light microscopy, but one long and one short flagellum are always observable under the electron microscope.

Furthermore, the distinctive features of the *Hydrurus* species studied were compared to further chrysophytes isolated from melting snow (Table S2, Table S3). *Hydrurus* species described from snow (this study) and *Chromulina ettlii* described from brownish snow in the High Tatras (Valley of the Zelene Lake, below the Medené lavky range, Slovakia; Hindák, 1969), *C*. cf. *elegans* reported from Mt. Phillistine snow, New Zealand (Novis, 2002a, 2002b) and spring cryoseston in Vitosha Mts, Bulgaria (Lukavský et al., 2009), and *Kremastochrysopsis austriaca* from the Austrian Alps (Remias et al., 2020) are similar in having one flagellum observable in a light microscope and in the shape of the chloroplast, which is parietal. However, they differ in the shape of cells: *C. ettlii* forms spherical cells, *C*. cf. *elegans* has spherical to ellipsoidal cells, and *K. austriaca* makes spherical to pyriform to oval cells. It must be noted that for *C. elegans* and *C. ettlii*, no genetic sequences are available, so it remains a question as to how they are connected. *Kremastochrysopsis austriaca* is a member of the Hibberdiales clade (Remias et al., 2020). *Hydrurus svalbardensi*s, *H. nivalis*, and *H. nemcovae* (this study) and *Chromulina chionophilia* described from mountain snow near Vancouver, British Colombia, Canada (Stein, 1963) were similar in cell shapes, forming ovoid, flattened flagellates in cross section, but they differed in a subtle morphological detail: The stigma of the former was prominent, not as tiny as in *C. chionophilia*. Another differentiating feature is the length of the flagellum (Hoham & Blinn, 1979). Last but not least, they are genetically different: *Hydrurus* species belong to Hydrurales, whereas *Chromulina chionophilia* is a member of the Chrysosaccales clade (Kristiansen & Škaloud, 2017). *Hydrurus novisii*, *H. klavenessii*, *H. tatrae*, *H. pulcher*, and *H. pascheri* (this study) and *Ochromonas smithii* described from snow in Japan (Fukushima, 1963) do not have stigma and develop tetrahedral flagellates, but the cell sizes of *O. smithii* are larger. *Hydrurus svalbardensi*s, *H. nivalis*, and *H. nemcovae* (this study) and *O. itoi* described from Japan (Fukushima, 1963) are similar in having stigma, but *O. itoi* is shorter in the length of the cells.

### Habitat descriptions

**FIGURE 9:**
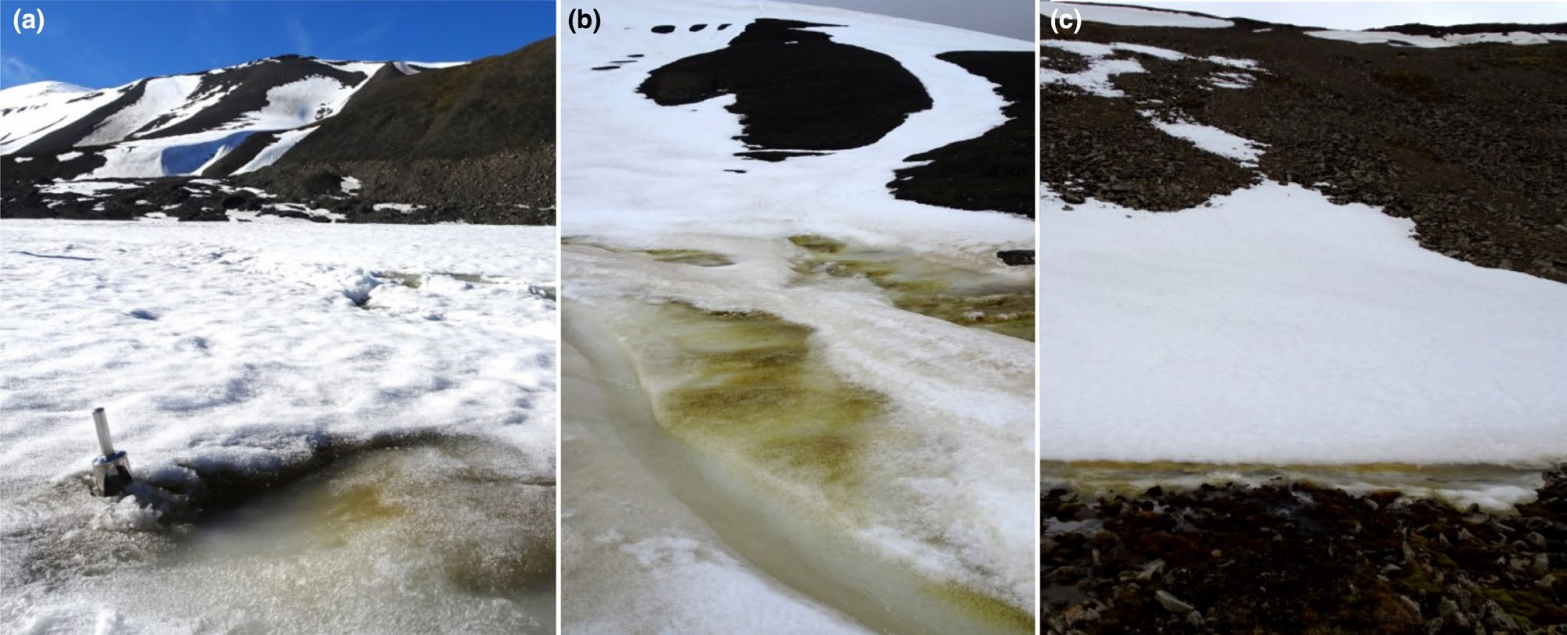
Overview of three selected sampling sites with golden‐brown snow blooms caused by new *Hydrurus* sp. on Arctic Svalbard, Norway. (a) cells below fresh snow in slush above the surface of a glacier rivulet (Longyearbreen, WP198), (b) several square meters with slushy discolorations adjacent to a glacial meltwater river (originating from Platåbreen, WP203), (c) slush stripe at the lower margin of a steep, rock‐bedded snow field in a narrow valley (upper Gruvedalen, WP201).

In the Alps, spots of golden‐brown snow were observed at mid‐June at altitudes from 1745 to 2147 m a.s.l. (Figure S3d–m, Table 1), while the Arctic ones were observed in early to mid‐July between 161 and 565 m a.s.l. (Figure 9, Figure S3n–p, Table 1). Scattered Dhrysophycean cells in a red bloom of *Sanguina* (Figure S3a) and a green bloom of *Chloromonas* (Figure S3b,c) were located at altitudes of 2379 and 2022 m a.s.l. (Table 1), respectively.

The meltwaters had a pH range of 5.7–5.9, and the electrical conductivity ranged from 8 to 47 μS · cm^−1^. Spots of golden‐brown blooms occurred commonly on the snow surface or slightly below, either faintly, as yellowish to golden‐brown in color, which was characteristic of the Alps, or more visually prominent at Svalbard, where in one case, large surficial blooms developed in waterlogged snow (uncultured *Hydrurus* sp.—WP203, Figure 9b). At some sites, the blooms were not directly evident because they developed deeper in the snowpack, for example, above a basal ice plate in the Alps (*H. nemcovae* WP271, Figure S3k–m), below fresh snow and above the surface of a glacier rivulet (Longyearbreen, uncultured *H*. sp. WP198, Figure 9a), or apparent as slush at the lower margin of a steep snow field (upper Gruvedalen, uncultured *H*. sp.‐WP201, Figure 9c).

The snow chemistry associated with golden‐brown snow blooms at the Alpine and Arctic sites is summarized in Table 3. Overall, ${\mathrm{NO}}_{3}^{-}‐N$ and ${\mathrm{NH}}_{4}^{+}‐N$ were the most abundant inorganic nitrogen forms in the Alps and Arctic, respectively. In one case, a very high total nitrogen value at one alpine site of uncultured *Hydrurus* sp. WP403 was caused by high ${\mathrm{NO}}_{3}^{-}‐N$ and ${\mathrm{NO}}_{2}^{-}‐N$, likely due to car traffic, because the sample was next to the mountain road. Similarly, high values of Na^+^ and Cl^−^ were reported from an Arctic site (*H. svalbardensis* WP301) and an alpine site next to a road (uncultured *H*. sp. WP403).

**TABLE 3:** Selected chemical characteristics of meltwater of golden‐brown blooms from one Arctic (WP301, *Hydrurus svalbardensis*) and three Alpine sites (WP395, WP401, WP403, uncultured *Hydrurus* spp.).

Sample ID	pH	EC, μS · cm^−1^	${\mathrm{NH}}_{4}^{+}‐N$, μg · L^−1^	${\mathrm{NO}}_{2}^{-}‐N$, μg · L^−1^	${\mathrm{NO}}_{3}^{-}‐N$, μg · L^−1^	${\mathrm{PO}}_{4}^{3-}‐P$, μg · L^−1^	Cl^−^, mg · L^−1^	Na^+^, mg · L^−1^	K^+^, mg · L^−1^	Ca^2+^, mg · L^−1^	Mg^2+^, mg · L^−1^	DOC, mg · L^−1^	IC, mg · L^−1^
WP 301	5.7	23	63	0.2	14	29	2.5	2.62	0.21	0.47	0.06	14.56	13.4
WP 395	5.7	8	10	1.0	29	21	0.9	0.48	0.1	0.49	0.02	2.3	0.93
WP 401	5.9	8	5	0.8	75	13	1.0	0.75	0.09	0.43	0.05	1.2	1.25
WP 403	5.9	47	25	7.1	463	22	2.7	2.15	0.47	4.91	0.58	7.26	2.76

*Note*: Chemical analyses—pH, Ca^2+^, Mg^2+^, Na^+^, K^+^, ${\mathrm{NH}}_{4}^{+}‐N$, ${\mathrm{NO}}_{2}^{-}‐N$, ${\mathrm{NO}}_{3}^{-}‐N$, ${\mathrm{PO}}_{4}^{3-}‐P$, dissolved organic carbon (DOC, mg · L^−^^1^), inorganic carbon (IC, mg · L^−^^1^).

### Population densities and cell morphologies

In nearly all cases, virtually monospecific Chrysophycean blooms made of motile cells were observed in the field, with two exceptions: Sample WP195 (*Hydrurus tatrae*) was mixed with green snow dominated by *Chloromonas* flagellates, and the sample used for isolation of *H. pulcher* was mixed with red snow dominated by *Sanguina*. A low number of *Chloromonas* cells were present in samples of *H. nivalis* (WP225) and *H. novisii* (WP264). The main constituents of the blooms were either Chrysophycean tetrahedral flagellates (uncultured *Hydrurus* sp.: WP198, WP201, WP203 and *H. klavenessii* WP222.2), a mixture of flagellates and the tetrahedral flagellates (*H. novisii* WP264, *H. pascheri* WP227), or stigma‐bearing swarmers (*H. nivalis* WP225). In some cases, very fast swimming flagellates and, more specifically, elongate‐shaped flagellates were noticed when using a field microscope, but cells quickly degenerated during observation, impeding a detailed morphological characterization (*H. nemcovae* WP271 and *H. svalbardensis* WP301). The maximum population densities observed were 8.9 × 10^5^ ± 0.44 × 10^5^ cells · mL^−1^ meltwater (*H. pascheri* WP227), 13.7 × 10^3^ ± 1.5 × 10^3^ cells · mL^−1^ meltwater (*H. novisii* WP264), 14.7 × 10^3^ ± 1.7 × 10^3^ cells · mL^−1^ meltwater (*H. nemcovae* WP271), and 4.1 × 10^6^ ± 0.22 × 10^6^ cells · mL^−1^ meltwater (*H. svalbardensis* WP301).

### Molecular phylogeny and ITS2 rRNA region

**FIGURE 10:**
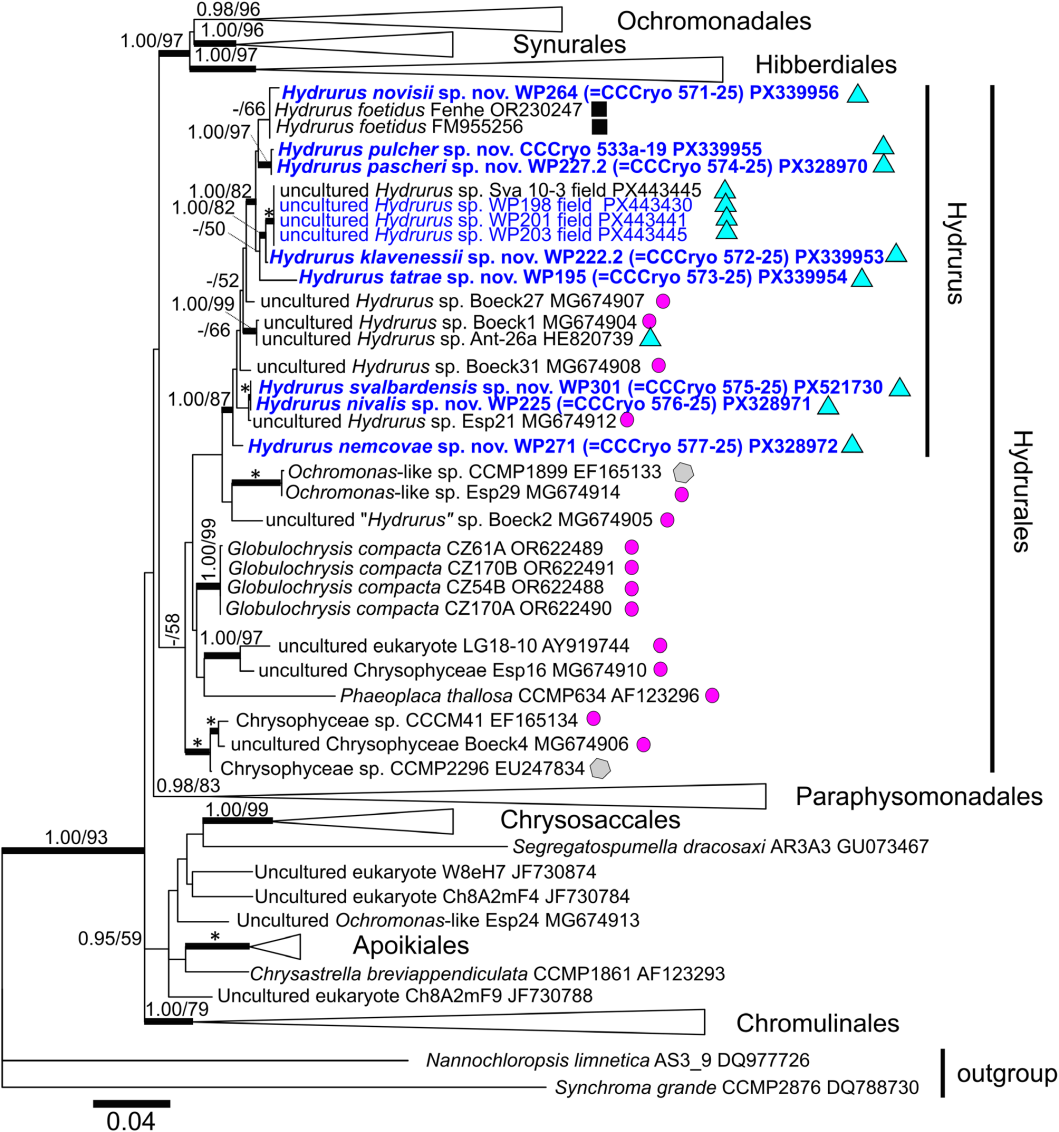
18S rRNA gene‐based maximum likelihood phylogenetic tree with the new sequences obtained from snow blooms in blue and the new strains in blue bold, respectively. Accession numbers, strain and field sample codes are indicated after each species name. The scale bar shows the estimated number of substitutions per site. Posterior probabilities (0.95 or more) and bootstrap values from maximum likelihood analyses (50% or more) are shown. Full statistical support (1.00/100) is marked with an asterisk. Thick branches represent nodes receiving the highest posterior probability support (1.00). Symbols in Hydrurales indicate sequence origin from strains/field samples: Either from snow (blue triangle), mountain brooks (black box), lakes (violet circles), or sea ice (gray polygon).

Based on the phylogeny of the 18S rRNA gene sequences (Figure 10), all golden‐brown field blooms and the isolated strains belong to a well‐supported clade *Hydrurus* (posterior probability/bootstrap value from maximum likelihood analyses: 1.00/87) within the Hydrurales clade. The only morphologically identified species in this subclade was the macroscopic *H. foetidus* sampled from a cold water stream in Finse, Norway (FM955256) and from colonies on stones in an alpine river in China (OR230247). The further closest relatives of the eight new species in molecular databases were reported as “uncultured snow algae” from both polar regions, Svalbard (Sva10‐3, HE820740) and Antarctica (Ant‐26a, HE820739). The next closest relatives in this subclade were uncultured *Hydrurus*‐like Chrysophyceae, as shown by sequencing of plankton samples from Antarctic lakes; these were observed in oligotrophic (Esp21, accession number MG674912) and mesotrophic waters (e.g., Boeck27, accession number MG674907).

The phylogeny based on *rbc*L gene data congruently confirmed the placement of the new strains in the Hydrurales clade (Figure S4). The deeper relationships in this clade, however, could not be resolved due to the current scarcity of available *rbc*L gene sequences for this clade.

The ITS2 rRNA region sequencing of the new strains revealed that each culture represented an independent entity, possessing a unique ITS2 rRNA region sequence. The only sister species, which had 100% matching 18S rRNA gene sequences in this study but a very different ITS2 rRNA region sequences, were the Arctic *Hydrurus svalbardensis* (WP301) and the Alpine *Hydrurus nivalis* (WP225). Additionally, ITS2 rRNA region sequencing of other snow fields around Longyearbyen in Svalbard revealed that the golden‐brown blooms there were caused by a further yet‐undescribed species of *Hydrurus*, which was locally widespread in the 2018 season (samples WP198, WP201, WP203). This alga was observed previously (sample Sva10‐3) in 2011, but no cultivation efforts were made; in this study we have called it “uncultured *Hydrurus* sp.”

### Light preferences of field populations

**FIGURE 11:**
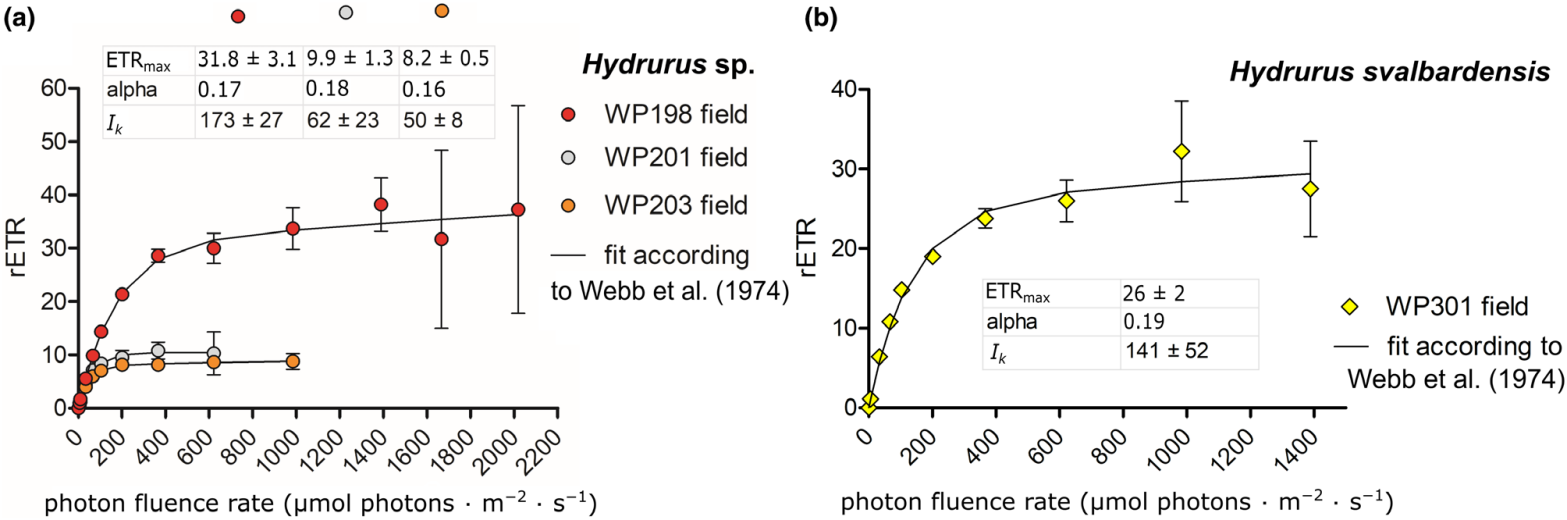
Photosynthetic rapid light curves of chrysophytes causing snow blooms on Svalbard (a) a performance of one still uncultured/undescribed *Hydrurus* species blooming at three different sites during early July 2018 (WP198, WP201, and WP203) (b) *Hydrurus svalbardensis* at the end of the melting season in the second half of July 2022 (WP301). The effect of increasing PAR (*x*‐axis) on the relative electron transport rate (*y*‐axis) in chloroplasts is shown. Values are means of four independent biological replicates (±SD).

Light‐dependent relative electron transport rates (ETRs) were measured at ambient temperature (field conditions) in the Arctic to reveal the extent of PSII adaptation to the habitat. The tested field communities of uncultured *Hydrurus* sp. (Figure 11a) and *H. svalbardensis* (Figure 11b) were photophysiologically active. They exhibited an α (graph slope in the light‐limited section of the photosynthesis‐irradiance curve; P‐I) of 0.17 (WP198), 0.18 ± 0.1 (WP201), 0.16 ± 0.2 (WP203), and 0.19 ± 0.02 (WP301). They had a relative ETR_max_ of 31.8 ± 3.1 (WP198), 9.9 ± 1.3 (WP201), 8.3 ± 0.5 (WP203), and 26 ± 2 (WP301). They showed *I*_k_ values of 173 ± 27 (WP198), 62 ± 23 (WP201), 50 ± 8 (WP203) and 141 ± 52 (WP301) μmol photons · m^−2^ · s^−1^. No signs of photoinhibition were noticed.

### Fatty acid composition

**FIGURE 12:**
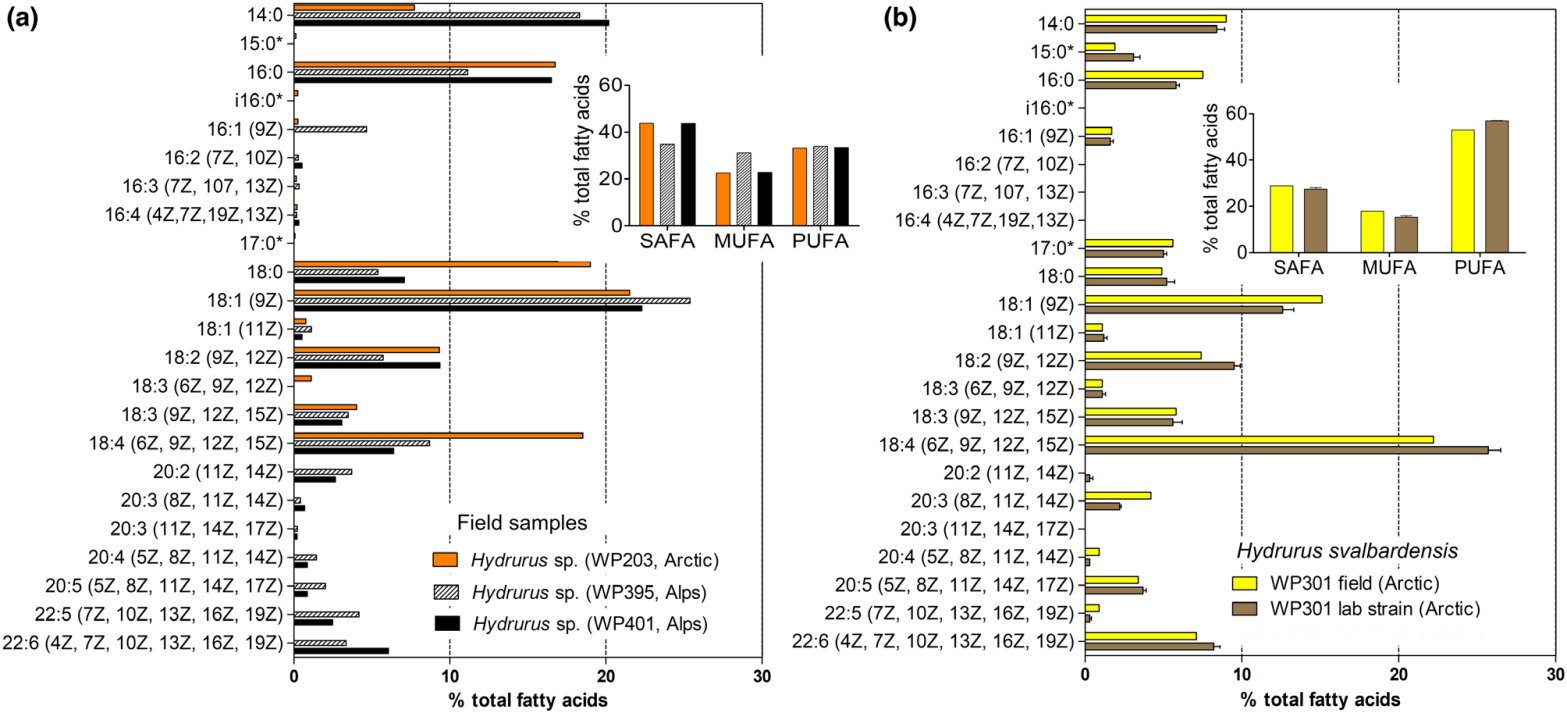
Mean cellular fatty acid composition of (a) field populations of *Hydrurus* sp. in the Arctic vs. *Hydrurus* spp. in the Alps, (b) *Hydrurus svalbardensis* sp. nov.: field (*n* = 1) vs. its lab strain grown at +1/−1°C (day/night; *n* = 3). Numbers in % of total fatty acids (±*SD*). The relative proportion of saturated (SAFA), monounsaturated (MUFA), and polyunsaturated (PUFA) fatty acids (±*SD*) is given in the inset. The figure shows only fatty acids with abundances greater than 0.2%, “*” bacterial contamination. A detailed fatty acid profile of all field samples and strains including FAs accounting at least for 0.1% in total fatty acids is in Table S4.

The relative content of fatty acids (FAs, in percentage of total FAs) of *Hydrurus* field cells and of one laboratory strain is shown in Figure 12. Fatty acids with chain lengths from C14 to C22 were found.

In case of *Hydrurus svalbardensis*, field (WP301 field, Arctic) versus laboratory strain (WP301 lab strain) were compared (Figure 12b): The samples showed high levels of PUFAs (53% vs. 56.87% ± 0.17% of total fatty acids), whereas the content of saturated fatty acids (SAFAs) did not exceed one‐third (28.9% vs. 27.43% ± 0.66%). The SAFAs were dominated by myristic acid (14:0): 9.0% versus 8.37% ± 0.49%. The main monounsaturated fatty acid (MUFA) was oleic acid (18:1; 9Z): 15.1% versus 12.6% ± 0.73%. The dominant PUFA was stearidonic acid (18:4; 6Z,9Z,12Z, 15Z): 22.2% versus 25.7% ± 0.78%, followed by linoleic acid (18:2; 9Z,12Z): 7.4% versus 9.53% ± 0.41%. Furthermore, the alga produced long chain PUFAs such as arachidonic acid (20:4; 5Z, 8Z, 11Z, 14Z): 0.9% versus 0.33% ± 0.05%, eicosapentaenoic acid (20:5; 5Z, 8Z, 11Z, 14Z, 17Z): 3.4% versus 3.7% ± 0.2%, and docosahexaenoic acid (22:6; 4Z, 7Z, 10Z, 13Z, 16Z, 19Z): 7.1% versus 8.5% ± 0.4%.

In the case of the polar field sample “undescribed *Hydrurus* sp.” (WP203) versus two high Alpine samples of *Hydrurus* spp. (WP395, WP401; Figure 12a), the SAFAs content (43.8% vs. 34.8% and 43.7%) was equal to or slightly higher than the PUFAs content (33.3% vs. 34% and 33.4%). The main MUFA was oleic acid (21.5% vs. 25.4% and 22.3%), and the dominant PUFA was stearidonic acid (18.5% vs. 8.7% and 6.4%). Long chain PUFAs were present in the following quantities: arachidonic acid (n/d vs. 1.45% and 0.86%), eicosapentaenoic acid (n/d vs. 2% and 0.9%) and docosahexaenoic acid (n/d vs. 3.4% and 6.1%).

**FIGURE 13:**
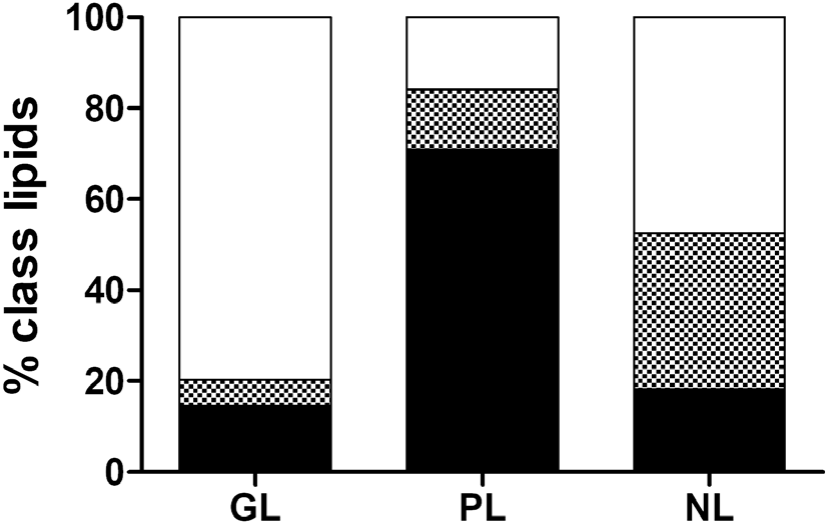
The relative proportions of saturated (white), monounsaturated (black‐white checked) and polyunsaturated fatty acids (black) in *Hydrurus* sp. from snow in the Arctic (field sample WP203) in the three main lipid classes (GL, glycolipids; NL, neutral lipids; PL, phospholipids).

Cellular neutral lipids predominated over glycolipids and phospholipids in total lipids in the *Hydrurus* sp. field sample (WP203; Table 4). Saturated FAs dominated glycolipids and were abundant in neutral lipids, whereas PUFAs dominated phospholipids in *Hydrurus* sp. field samples (WP203; Figure 13).

**TABLE 4:** Relative amounts of lipid fractions in *Hydrurus* sp. (WP203) field sample. Values are in (%) of total lipids.

	*Hydrurus* sp.
Neutral lipids	51.0
Glycolipids	19.0
Phospholipids	30.0

### Pigment composition

The chromatographic pigment analysis of *Hydrurus pulcher* (CCCryo 533a‐19) showed compounds typical for the chloroplasts of chrysophytes, namely Chl *a* and Chl *c*, the primary carotenoids β‐carotene and fucoxanthin and, in addition, a series of xanthophyll‐cycle pigments: violaxanthin, antheraxanthin, and zeaxanthin (Table 5).

**TABLE 5:** Pigment composition per dry matter of *Hydrurus pulcher* strain CCCryo 533a‐19 after incubation at 3°C in day:night regime of 16:8 h at 13–16 μmol photons · m^−2^ · s^−1^.

Pigment	Concentration (mg · g^−1^ dry matter) ± *SD*
Chlorophyll *a*	15.98 ± 0.09
Chlorophyll *c*	0.28 ± 0.02
Fucoxanthin	10.2 ± 0.12
Violaxanthin	4.09 ± 0.01
Antheraxanthin	0.17 ± 0.01
Zeaxanthin	0.13 ± 0.01
β‐Carotene	0.53 ± 0.01
Sum unidentified pigments	0.7 ± 0.23

*Note*: Values are means of two independent biological replicates (±*SD*).

### Sugar alcohol as antifreeze substance

Based on chromatography of aqueous extracts, all analyzed *Hydrurus* strains showed peaks with retention times typical for sugar alcohols (polyols), which likely act as cytosolic compatible solutes (osmoprotectants). Two of them were identified as arabitol and sorbitol, and the cellular contents were quantified (Table 6). *Hydrurus klavenessii* showed the highest proportion, with more than 0.5% per algae dry matter.

**TABLE 6:** Quantitative data of sugar alcohols arabitol and sorbitol in cells of tested *Hydrurus* strains, measured by means of HPIC.

Species/compound	Arabitol, μg · mg^−1^ DW	Sorbitol, μg · mg^−1^ DW
*H. klavenessii* CCCryo 572–25	1.259	3.806
*H. novisii* CCCryo 571–25	3.950	1.092
*H. nivalis* CCCryo 576–25	n.d.	n.d.
*H. svalbardensis* CCCryo 575–25	0.673	0.185
*H. nemcovae* CCCryo 577–25	0.903	n.d.

*Note*: Further chromatographic peaks with according retention times for polyols were present but not identified.

Abbreviation: n.d., not detected.

## DISCUSSION

### Recent taxonomic advances related to cold‐adapted Chrysophyceae

Malavasi et al. (2024) described the current taxonomic situation of Chrysophycean algae. On the one hand, environmental sequencing indicated a high cryptic diversity, but on the other hand, Malavasi et al. (2024) claimed that traditional, morphologically defined genera proved to be polyphyletic and, moreover, rich in undescribed genera (i.e., *Ochromonas‐*like flagellates). In the green algae order Chlamydomonadales, both genera and species living in snow developed independently several times in evolution (Novis et al., 2024). This must also have happened in the Chrysophyceae because cryophilic species have been observed within the Hibberdiales (Remias et al., 2020), Chromulinales (Soto et al., 2020), and Hydrurales (this study). The latter order comprised four morphologically distinct genera. First, the until now monospecific *Hydrurus foetidus* formed macroscopic thalli growing in cold freshwater and was distributed approximately beyond a 40° N to 40° S latitudinal belt (Klaveness, 2019). Second, the pseudo‐parenchymatous marine *Phaeoplaca* (Kristiansen & Škaloud, 2017), and third, *Globulochrysis*, with closely packed cells developing into large, spherical colonies in freshwater (Malavasi et al., 2024). Finally, the morphologically *Ochromonas*‐like flagellates isolated from Antarctic sea ice, such as CCMP1899.

### Taxonomic reassignment of snow chrysophytes from *Ochromonas* to *Hydrurus*

In the snow of the South Orkney Islands in Antarctica, one of the most abundant algal species was identified as related to, or possibly identical with, *Ochromonas itoi* and *O. smithii* (Fogg, 1967), which both were described from Japanese snowfields (Fukushima, 1963) as the first records of Chrysophyceae as a cryophilic alga. Half a century later, the golden snow observed on Mt. Gassan, a beech forest (deciduous forest) in Japan, was attributed to *O. itoi* and *O. smithii* (Tanabe et al., 2011). Both algae were traditionally identified by their morphologies (Tanabe et al., 2011) and later sequenced, resulting in the conclusion that both algae formed a clade together with *Hydrurus foetidus* and that this clade was independent from many other species of *Ochromonas* (Shitara et al., 2009). But the sequence data remain unavailable (Ryo Matsuzaki, pers. comm.). *Ochromonas smithii* motile cells were reported to be tetrahedral in shape, whereas *O. itoi* flagellated cells were described as laterally kidney‐shaped, triangular, rhomboid, or pear‐shaped (Fukushima, 1963). Since *O. smithii* possesses characteristic tetrahedral flagellate stages, it likely belongs to *Hydrurus*. In this study, we confirmed with eight new species that the studied *Chromulina*‐like and *Ochromonas*‐like flagellate morphologies living in melting snow are assignable to the genus *Hydrurus*. Consequently, *O. itoi* may also belong to *Hydrurus*. The high number of *Ochromonas*‐annotated sequences reported from the cryosphere in earlier metabarcoding studies needs to be reevaluated, as was the case when Remias et al. (2013) claimed that *Hydrurus*‐related unicells caused snow blooms in polar coastal regions. This situation aligns well with our finding that the unicellular *H. novisii* differs from macroscopic *H. foetidus* in its 18S rRNA gene by only 1 bp (out of 1687 bp), whereas their ITS2 rRNA regions are clearly different (90% sequence similarity, out of 256 bp).

### Preferred habitats of *Hydrurus* species

The typical habitats of *Hydrurus foetidus* are streams fed by glaciers, subterranean ice, seasonal snowpacks (Jorgenson et al., 2024), and other oligotrophic environments with high light availability and rapidly moving waters such as cold springs in the Alps (Cantonati et al., 2006). According to Bursa (1934), Kann (1978) and Canter‐Lund and Lund (1995), thalli begin to decay when the water temperature rises above 16°C during summer; as a consequence, some cells transform into cysts, many probably die, and the thalli disappear.

In the case of the snow‐dwelling unicellular *Hydrurus*, some species prefer waterlogged ice slush where blooms occur at the interface between the snow surface and a solid ice plate below (likely caused by underground permafrost in polar regions; Remias et al., 2013). Others thrive under fresh, melting snow or near supraglacial rivulets (see *Hydrurus* sp. in Figure 9). Noteworthy, other *Hydrurus* species were observed in drier melting snowpacks (*H. nemcovae*); however, they were observed to have lower cell concentrations than species dwelling in slushy sites. Addressing the dynamic nature of snowpack habitats, the distribution and abundance of *Hydrurus* blooms are likely controlled by the rapid flux of liquid water, available nutrients (from the snowpack and meltwater chemistry), and light conditions. The high cell concentrations observed in slushy sites suggest that ample liquid water availability is a major factor promoting large blooms. In the presence of an ice plate in a snowpack, the cells were distributed horizontally along this plate (e.g., *Hydrurus nemcovae* WP271). Furthermore, these chrysophytes exhibited an adaptive photophysiology capable of thriving across a spectrum of irradiances, from the very low light typical of deeper snow or lower light on the snow surface during foggy days to the high irradiation encountered on the snow surface during a sunny day in the melting season (this study). Although the ecophysiological mechanisms enabling such rapid colonization remain unknown, these factors collectively influence species success as the snowpack changes. Sequencing revealed that some *Hydrurus* species inhabit slush, melting snowpacks (this study) and cryoconite holes (Zawierucha et al., 2022) whereas others are among the phytoplankton of polar (Izaguirre et al., 2021) or temperate lakes (Bock et al., 2022). Many Chrysophyceaen taxa originating from snow were observed to remain in the potentially active fraction (rRNA data) of downstream microbiomes in the hydrological continuum of the Canadian Arctic (Comte et al., 2018). Ongoing climate change may have an impact on this connectivity. Conversely, there was a recent discovery of an unexpected non‐obligate physical association between *H. foetidus* and *Paralemanea* (Rhodophyta), in which *H. foetidus* was repeatedly observed to grow as an epiphyte on thalli in Serbia. This suggests an adaptation to survive the transiently elevated water temperatures in rivers during summer, with the temperature record of *H. foetidus* occurring at a water temperature that varied between 7.3°C and 18.9°C (Rakonjac & Simić, 2024).

### Chemical characteristics of the melting snowpack

Chemical snow characteristics of golden‐brown blooms resembled those of red snow communities regarding the relatively low phosphorus conditions (${\mathrm{PO}}_{4}^{3-}$ = 0.53 μM, i.e., 16.4 μg · L^−1^ ${\mathrm{PO}}_{4}^{3-}‐P$; in green snow it was one magnitude higher, Lutz et al., 2015). A golden‐brown snow bloom from the coastal Arctic was similar to green snow communities in elevated dissolved organic carbon (1192 μM, i.e., 14.3 mg · L^−1^, Lutz et al., 2015), whereas the golden‐brown blooms from the Alps were in either two times or one time magnitude lower dissolved organic carbon. Interestingly, high dissolved organic carbon was a significant predictor of chrysophyte cyst distribution in the Adirondack lakes (Duff et al., 1997). This may reflect that some aquatic chrysophytes are strongly dependent on ingesting bacteria for nutrition rather than being dependent solely on photosynthesis (Bird & Kalff, 1987). In snow blooms in the Alps, the dissolved organic carbon may come mainly from the algae themselves during their life and after they decay, but in coastal regions, airborne local sources of dissolved organic carbon should also be considered (e.g., large bird colonies nesting on the rock of *Alle alle*). Concerning ${\mathrm{NO}}_{3}^{-}‐N$, one golden‐brown snow sample was nutrient rich, whereas all other samples showed moderate nitrogen availability (dozens of micrograms per liter of ${\mathrm{NO}}_{3}^{-}‐N$), similar to that reported for green snow (${\mathrm{NO}}_{3}^{-}$ = 296 ppb, i.e., 67 μg · L^−1^ ${\mathrm{NO}}_{3}^{-}‐N$; Lutz et al., 2015). Higher Na^+^ and Cl^−^ concentrations in one snow community on Svalbard and one in Austria may be a result of sea spray (Domine et al., 2004) and due to de‐icing salts spread on alpine roads during wintertime (Niedrist et al., 2021), respectively. The values measured during the blooms reflect abiotic characteristics when samples were taken, but do not explain why a bloom can develop at a certain site earlier in the season. Ideally, these parameters should be measured from the beginning of snowmelt in a time series. The appearance of an algal bloom on a snow surface is likely a balance between the duration of the melt and the timing of a new snowfall event (Onuma et al., 2022). So far, it is evident that the availability of liquid water is important for bloom development (Remias, 2012).

### *Hydrurus* cell morphologies in relation to abiotic parameters

Depending on the prevailing environmental conditions, morphological plasticity was observed in all described *Hydrurus* species exhibited morphological plasticity under varying environmental conditions: At around 0°C, flagellates were very motile; however, when *Hydrurus novisii* was grown at 15°C, cells entered into immotile stages. The latter temperature was lethal for *H. pascheri*. Accordingly, at around 0°C, *Hydrurus* swarmers from snow exhibited a swimming speed of about 22 μm · s^−1^ (Détain et al., 2025), comparable to cryoflora taxa of the green algae, such as flagellates of *Sanguina nivaloides* (about 20 μm · s^−1^) and *Chloromonas hindakii* (about 19 μm · s^−1^). Vital motility is advantageous in rapidly changing light conditions, as it enables optimal irradiation levels to be reached quickly within the day. Consistently, golden‐brown blooms were observed on the snow surface only during overcast or foggy conditions in both the Alps and the Arctic. In contrast, on sunny days, the golden‐brown algal community was observed in deeper parts of the snowpack.

The tetrahedral flagellate stages of *Hydrurus foetidus* are well documented (Hoffman et al., 1986; Klebs, 1893). Cells of this type, as well as pentahedral and hexahedral flagellates, were observed in *H. klavenessii* (WP222.2). The position of these unusual polyhedral flagellates within their life cycle is unclear. Their role as putative planozygotes was discussed earlier (Remias et al., 2013). Given that some of the studied strains often or permanently generate these stages abundantly, albeit to varying extents depending on the species, an alternative hypothesis must be considered. For example, these stages may be an adaptation to a life in floating waters, as they are zoospores. The non‐motile, attached stages of *H. foetidus* release motile single cells when they are ready to colonize new locations or to reproduce (Hoffman et al., 1986). Such cells possess this characteristic shape. Transmission electron microscopy revealed that the cell shape of these motile stages in *H. foetidus* is maintained by a complex microtubule‐based skeletal system (Hoffman et al., 1986), which is remarkably similar to the ultrastructural details of *Chrysonebula holmesii* (Hibberd, 1977). *Chrysonebula holmesii* also lives as macroscopic colonies attached to stones in streams (Lund, 1953). Loosely developed Chrysophycean colonies (i.e., individual cells or two to four cells arranged peripherially in joint mucilage) on stones in Europe were reported also for *Celloniella palensis* (Pascher, 1929). Based on material from Australia, observations of *Celloniella palensis* showed that the zoospore initially was elongated. However, as it extended and repositioned its roots, it became triangular in shape. (personal observation). However, there is no molecular sequence record, and an assumed close relatedness of *Celloniella* to *Hydrurus* remains to be proven.

It is unknown whether the eight new species are able to grow when not within melting snow. If so, they should be regarded as facultative snow algae (cryotrophic). Additionally, it cannot be excluded that some of them would form macroscopic thalli when growing in habitats other than snow. We occasionally observed macroscopic cell aggregates in older cultures when grown in shaken or aerated liquid medium, but never on agar plates.

### Ultrastructure of flagella, flagella position

With TEM, the flagellates showed the typical combination of a short mature flagellum with a long immature flagellum possessing mastigonemes. The length of the shorter flagellum differed between *Hydrurus* species, being more prominent in *H. klavenessii* (WP222.2) than in *H. nivalis* (WP225).

### Stomatocysts and a hypothesis of recolonization of the bloom localities

The stomatocysts of *Hydrurus foetidus* have very distinctive wings (Klebs, 1893, figure plate XVIII, figure 19a–19d). These wings might be advantageous in rapidly flowing rivers. In this study, stomatocysts (resting stages of Chrysophyceae) of some *Hydrurus* species from snow, for example, of *H. klavenessii* and *H. nivalis*, had a low collar and did not show any ornamentations (no spines, no ridges). However, such stomatocysts were observed only in cultures in very low numbers, so we could make no statistical evaluation of their cell sizes nor include such an observation in the species descriptions. The absence of ornamentation or collar, resulting in a smooth cell surface, is a morphological feature seen in other common snow algae (*Sanguina nivaloides*; Ezzedine et al., 2023). This morphology and the small cell size could represent an adaptation for efficient long‐distance air dispersal (anemochory), facilitating colonization of new snow habitats. Although our culture medium was silica replete, it is not impossible that these smooth stomatocysts represent immature, poorly silicified cell stages, consistent with experimental observations that the complex ornamentation in chrysophytes is a late developmental feature and that poorly formed structures result from incomplete silicification (for stomatocysts: Siver, 1991; for scales: Sandgren et al., 1996). Strikingly, stomatocysts were never observed in field samples, which raises questions about the fate of the population after complete snowmelt. However, it must be noted that a low rate of chrysophyte cyst production, especially in asexual populations, has been documented in the literature (Holen & Princiotta, 2023; Sandgren, 1981), which might explain their absence in field samples. Can a local reservoir of dormant cells resting on soil, rock surfaces, or glacier ice be the source for the next melting/growth season? Such life cycles have been discussed for green algae living in snow (Remias, 2012). Furthermore, it is currently assumed that an inoculation of a pristine snow with snow algae developing into green or red bloom over the course of the season can occur in other ways: either by air or by being flushed by melting water onto snow and ice from locations above. One may wonder how it happens that the cysts arrive from higher mountain areas? Müller et al. (2001) hypothesized that at the end of the snow melting season, a dispersal of red snow algal cysts from dry snow, rock, or permafrost by wind into higher mountain areas takes place and that these populations inoculate suitable snow fields with the meltwater containing such algal cysts in next year's season. In case of *Hydrurus*, the latter (meltwater flushing) hypothesis was discussed in Rakonjac and Simić (2024) based on data from Cantonati et al. (2016). They showed the high importance of rheocrenes (spring resulting in a brook, neither pool nor seep) for a successful recolonisation of *Hydrurus* on epipelic substrates in brooks.

### Distribution of snow dwelling *Hydrurus* across the globe

The identity and robust phylogeny of key *Hydrurus* species were established using their 18S and 28S rRNA gene sequences from lotic chrysophyte *Hydrurus foetidus* (Klaveness et al., 2011). Later, based on (meta‐)genomic studies, 18S rRNA gene sequences assignable to the *Hydrurus* genus were common from cryohabitats. For instance, they were observed in golden‐brown snow at Svalbard (Remias et al., 2013) and in orange or red snow from Ardley Island at King George Island, Maritime Antarctica (Soto et al., 2020). Further localities were on Icelandic glaciers (Langjökull, Vatnajökull and Snæfallsjökull), showing that *Hydrurus* is common there during summer in cryohabitats such as snow, ice, the snow‐ice interface, and pro‐glacial water (Winkel et al., 2022). Yet, the absence of *Hydrurus* ITS2 rRNA region sequences in all the above‐mentioned research studies is most likely due to primers not targeting any chrysophytes. In general, care has to be taken for biodiversity evaluation at the species level when using sections of the 18S rRNA gene. In such a case, it is advisable to report on community compositions at the genus level only. In the future, the development of optimized ITS2 rRNA region primers will enable the identification of sequences with sufficient resolution at the species level within *Hydrurus*. This study provides ITS2 rRNA region sequences based on cultured material, which will facilitate the design of suitable primers for Illumina sequencing.

### Pigments and photosynthesis

Although cysts of Xhlamydomonadacean snow algae accumulate reddish astaxanthin in cytosolic lipid globules serving as photoprotection (Leya et al., 2009), the vegetative cells of snow chrysophytes abundantly produce plastidal fucoxanthin, which causes the overall golden‐brown color of the cells. In this study, the pigment composition of *Hydrurus pulcher* corresponded to those reported earlier for chrysophytes with tetrahedral cells from golden‐brown snow at Mt. Gassan in Japan, characteristically including two kinds of xanthophyll cycles for an effective dissipation of excessive irradiation (Tanabe et al., 2011). Accordingly, in vivo fluorimetry indicated that a tolerance against high light conditions exists, because no photoinhibition was noticed up to 1389 μmol photons · m^−2^ · s^−1^ (*H. svalbardensis*) or even up to 2018 μmol photons · m^−2^ · s^−1^ (*Hydrurus* sp.). Considering that ambient light conditions on Svalbard glaciers during sunshine were reported to reach 1104 μmol photons · m^−2^ · s^−1^ only (Remias et al., 2012), the PSII of these algae apparently is well adapted to high light conditions, even to the PAR values as tested, which have never occurred during polar summer in the field. Moreover, Chrysophyceae possess mycosporine‐like amino acids (MAAs) protecting against UVB (Kitzing et al., 2014). In a survey of 26 lakes in the Tyrolean Alps and Pyrenees, the highest total MAA concentration of 22.92 μg · L^−1^ was calculated in the chrysophyte‐dominated plankton of Mittlere Plenderlesee (Laurion et al., 2002). However, we found no MAAs in strains of this study (data not shown). The snow algae related to our species were shown to utilize the efficient violaxanthin cycle for photoprotection as a dissipation system of surplus energy under prolonged high‐light stress (Tanabe et al., 2011). However, fucoxanthin in snow chrysophytes is accumulated in larger amounts under low light conditions (personal observation), as it serves as a kind of antenna for the light‐harvesting complexes, whereas under high light laboratory conditions, they were reported to lose much of their carotenoid pigment, and the entire culture was pale green (Sutton, 1970). We assume that flagellated forms, such as those of *Hydrurus* species in snow, facilitate active movement within the water‐saturated snowpack. This mechanism allows them to optimize light exposure, and their migratory patterns reflect the weather/light conditions (e.g., cells move deeper to protect themselves from excessive solar radiation under sunny days or toward the surface to maximize light harvesting under cloudy/foggy days).

### Adaptation of metabolites, fatty acids, and polyols

A snapshot of the fatty acid profile of *Hydrurus foetidus* kept as a culture at 2.6°C and about 100 μmol photons · m^−2^ · s^−1^ (Klaveness, 2017) showed occurrences of steriadonic acid 18:4 (6Z, 9Z, 12Z, 15Z; 16.5%) and myristic acid (27.3%), and these numbers were comparable with the snow‐dwelling *Hydrurus* in our study. However, the long‐chain PUFAs arachidonic acid (0.3%), eicosapentaenoic acid (1.8%), and docosahexaenoic acid (3.6%) were present in two‐ to fourfold lower quantities in *H. foetidus* (Klaveness, 2017) compared to *Hydrurus* from snow (this study). The PUFAs dominated the FA pool in *H. svalbardensis* but were less than 50% of all FAs in both *H. foetidus* (Klaveness, 2017) and *Hydrurus* sp., indicating that PUFAs play an important role in maintaining membrane fluidity in cold environments (Morgan‐Kiss et al., 2006), yet their individual proportion relative to all FAs differed between species. The observed predominance of cellular neutral lipids over glycolipids and phospholipids in the *Hydrurus* field sample (WP203) suggests that the algae may be accumulating energy reserves, potentially as a response to environmental conditions such as nutrient availability or stress. The observed dominance of SAFAs within glycolipids and their higher abundance in neutral lipids further indicate a possible adaptation to maintain membrane stability under varying temperature regimes. Similarly, dominant SAFAs in glycolipids were reported for a snow alga *Sanguina aurantia* field sample from the high Arctic (Procházková et al., 2021). Additionally, the prevalence of PUFAs in phospholipids aligns with their role in maintaining membrane fluidity. A further ecophysiological strategy of *Hydrurus* from snow, to prevent low temperature damages, is the intracellular accumulation of sugar alcohols. Among these, glycerol was previously reported as abundant in *Hydrurus* from snow (Remias et al., 2013). Glycerol stabilizes biomembranes, consequently limiting possible damage by intracellular water stress during repeated freeze–thaw events. In other microalgae, arabitol and sorbitol were produced in response to osmotic stress (Gustavs et al., 2010), and we detected both polyols in some of our species as well.

## CONCLUSIONS

An unexpectedly high number of previously undescribed unicellular *Hydrurus* species were observed living in the melting snow of alpine and polar regions. They contribute to the biodiversity and productivity of cold terrestrial ecosystems, which are otherwise poor in these aspects. ITS2 rRNA region sequences of the algae causing golden‐brown snow provided sufficient resolution for the recognition at species level. In both their ecological role and genetic distinctness, they resemble blooms of green or red snow caused by green algae; however the complete life cycle, including resting stages as seeds for the next thawing season, remains unknown. Their closest relatives live in cold mountain streams and as cold freshwater lake plankton, suggesting that chrysophytes from low temperature aquatic and cryosphere habitats are ecologically and taxonomically closely related. The role of mixotrophy in quickly establishing these ephemeral blooms remains to be tested. These chrysophytes might rely on osmotrophy—the uptake of dissolved small organic molecules (e.g., organic acids or phenols) released during the initial snow melt. Such a strategy could provide a competitive advantage, allowing these species to outcompete others and form blooms rapidly. Although neither phagocytosis nor intracellular bacteria were observed via TEM in this study, the possibility of predatory activity cannot be entirely ruled out. Future research is needed to determine whether, to what the extent, and in what circumstances these *Hydrurus* species utilize various organic resources (Cormier et al., 2022).

## AUTHOR CONTRIBUTIONS

**Lenka Procházková:** Conceptualization (equal); data curation (lead); formal analysis (equal); funding acquisition (supporting); investigation (lead); resources (equal); visualization (lead); writing – original draft (lead); writing – review and editing (lead). **Robert A. Andersen:** Investigation (equal); visualization (equal); writing – review and editing (equal). **Thomas Leya:** Formal analysis (equal); investigation (equal); visualization (equal); writing – original draft (supporting); writing – review and editing (equal). **Tomáš Řezanka:** Formal analysis (equal); investigation (equal); writing – review and editing (supporting). **Martin Lukeš:** Formal analysis (equal); investigation (equal); writing – review and editing (supporting). **Linda Nedbalová:** Funding acquisition (equal); writing – review and editing (supporting). **Daniel Remias:** Conceptualization (equal); funding acquisition (equal); investigation (equal); resources (lead); writing – original draft (equal); writing – review and editing (equal).

## FUNDING INFORMATION

This research was funded by the Charles University Research Centre program No. 24/SCI/006 to LP, the Czech Science Project (GAČR) 24‐10019S to LP and LN, the Austrian Science Fund (FWF) P34073 to DR, and by the Institutional Research Concept RVO 61388971 (Institute of Microbiology, Prague, Czech Republic), Czech Ministry of Education (project PHOTOMACHINES, CZ.02.01.01/00/22_008/0004624).

## Supporting information


**Figure S1.** Sampling locations of unicellular *Hydrurus* spp. dwelling in melting snow (yellow circles). (a) Swiss Alps, (b) High Tatras in Slovakia, (c) around Longyearbyen in archipelago of Svalbard in Norway (a map extraction, courtesy of Norwegian Polar Institute, retrieved from http://toposvalbard.npolar.no on 21.07.2025), (d) Hohe Tauern (e) and Schladminger Tauern (d) in Austria. Numbering corresponds to the sample origin (ordered by snow sampling date). Habitat description of localities including geographical data are shown in Table 1.

**Figure S2.** Cell widths and cell lengths of vegetative cells of eight new species of *Hydrurus* isolated from snow (*n* = 30 for each strain). Note: the boxplot is drawn from quartile 1 to quartile 3 with a horizontal line drawn in the middle to denote the median. Whiskers (vertical lines) indicate actual minimum and maximum values in the dataset.

**Figure S3.** Field view of the type localities (highlighted by shovel or/and with arrow) of the eight new *Hydrurus* snow dwelling species described based on newly established algal strains in the course of this study: (a–m) Central Europe, (n–p) high Arctic. In detail, (a) *H. pulcher* CCCryo533a‐19, (b, c) *H. tatrae* WP195, (d, e) *H. klavenessii* WP222.2, (f) *H. nivalis* WP225, (g, h) *H. pascheri* WP227 (i, j), *H. novisii* WP264, (k–m) *H. nemcovae* WP271, (n–p) *H. svalbardensis* WP301. Order of the strains corresponds to the date of cryoflora sampling. Habitat description of localities including geographical data are shown in Table 1.

**Figure S4.** Bayesian phylogenetic tree based on the *rbc*L gene. The newly obtained sequences from snow algal strains are in blue bold. Accession numbers, strain and field sample codes are indicated after each species name. The scale bar shows the estimated number of substitutions per site. Posterior probabilities (0.95 or more) and bootstrap values from maximum likelihood analyses (50% or more) are shown. Full statistical support (1.00/100) is marked with an asterisk. Thick branches represent nodes receiving the highest posterior probability support (1.00).

**Table S1.** Recipe for Chrysophycea medium (DY‐V).

**Table S2.** Comparison of vegetative cells of six species of chrysophytes isolated so far from melting snow (*C*., *Chromulina*; *K*., *Kremastochrysopsis*; *O*., *Ochromonas*).

**Table S3.** Comparison of vegetative cells of the eight species of *Hydrurus* isolated from melting snow.

**Table S4.** Cellular fatty acid composition of *Hydrurus* (*H*.) sp. field samples (*f*) (WP203, WP395, and WP401), *Hydrurus svalbardensis* field (*f*) and strain (*s*) samples (WP301) in [%] of total fatty acids (TL; all samples) and in [%] of the three major lipid classes: Neutral lipids (NL), phospholipids (PL), and glycolipids (GL). The table shows only fatty acids that had abundances greater than 0.1%, “*” bacterial contamination. The relative proportion of saturated (SAFA), monounsaturated (MUFA), and polyunsaturated (PUFA) fatty acids is also given. Values of WP301 strain are means of three independent biological replicates (±SD).
