# Supplementary material for: Novel Hydrurus species (Chrysophyceae) and their adaptations to high‐altitude European and Arctic snowfields
Source: J Phycol. 2026 Apr 29;62(3):818–45. doi: 10.1111/jpy.70162 (PMC13280783; doi:10.1111/jpy.70162)
Supplement: Supplementary file 1 — Figure S1. Sampling locations of unicellular Hydrurus spp. dwelling in melting snow (yellow circles). (a) Swiss Alps, (b) High Tatras in Slovakia, (c) around Longyearbyen in archipelago of Svalbard in Norway (a map extraction, courtesy of Norwegian Polar Institute, retrieved from http://toposvalbard.npolar.no on 21.07.2025), (d) Hohe Tauern (e) and Schladminger Tauern (d) in Austria. Numbering corresponds to the sample origin (ordered by snow sampling date). Habitat description of localities including geographical data are shown in Table 1. [file JPY-62-818-s003.docx]

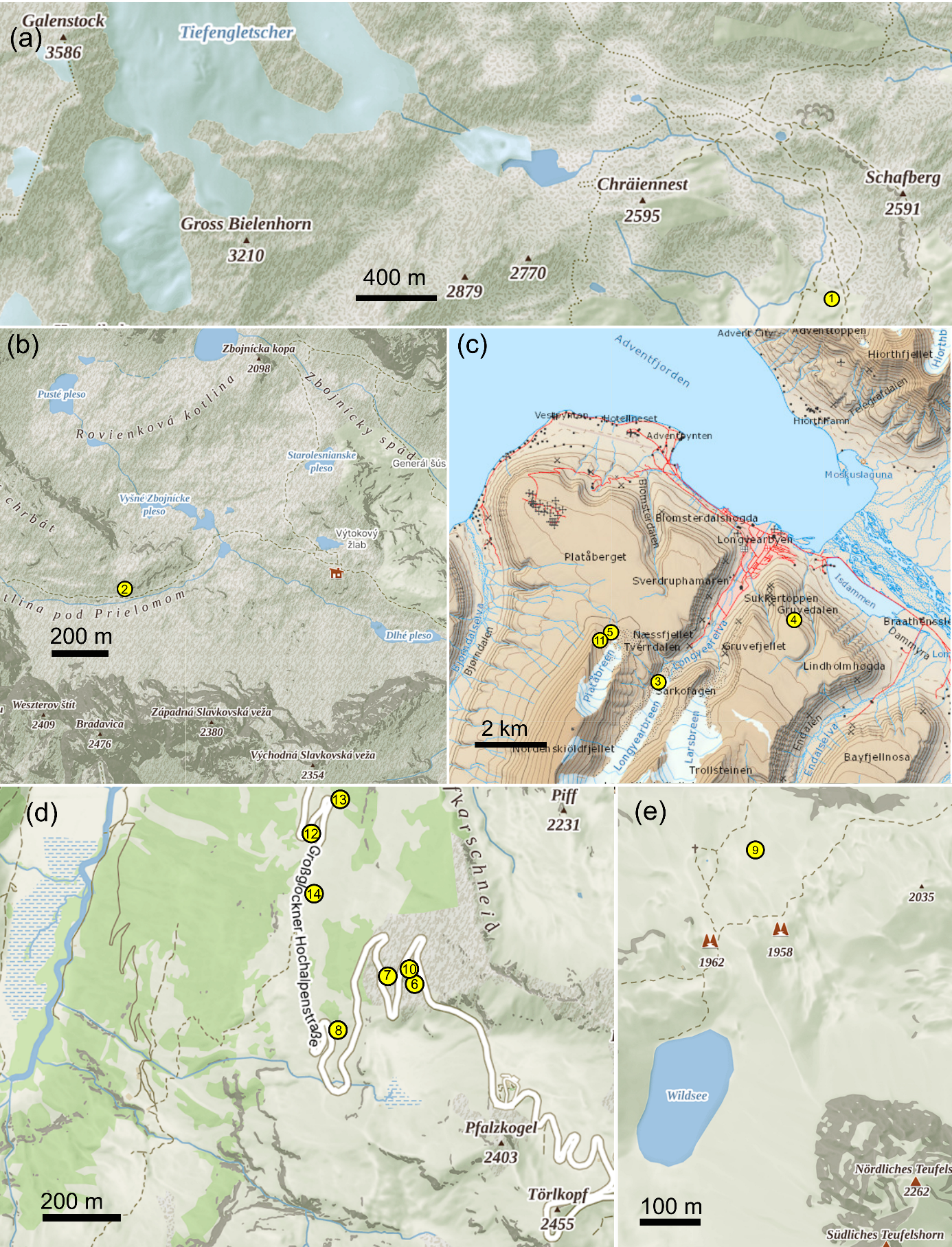


**Figure S1.** Sampling locations of unicellular *Hydrurus* spp. dwelling in melting snow (yellow circles). (a) Swiss Alps, (b) High Tatras in Slovakia, (c) around Longyearbyen in archipelago of Svalbard in Norway, (d) Hohen Taurn (e) and Schladminger Tauern (d) in Austria. Numbering corresponds to the sample origin (ordered by snow sampling date). Habitat description of localities including geographical data are shown in **Table 1**.
