# Supplementary material for: Novel Hydrurus species (Chrysophyceae) and their adaptations to high‐altitude European and Arctic snowfields
Source: J Phycol. 2026 Apr 29;62(3):818–45. doi: 10.1111/jpy.70162 (PMC13280783; doi:10.1111/jpy.70162)
Supplement: Supplementary file 2 — Figure S2. Cell widths and cell lengths of vegetative cells of eight new species of Hydrurus isolated from snow (n = 30 for each strain). Note: the boxplot is drawn from quartile 1 to quartile 3 with a horizontal line drawn in the middle to denote the median. Whiskers (vertical lines) indicate actual minimum and maximum values in the dataset. [file JPY-62-818-s008.docx]

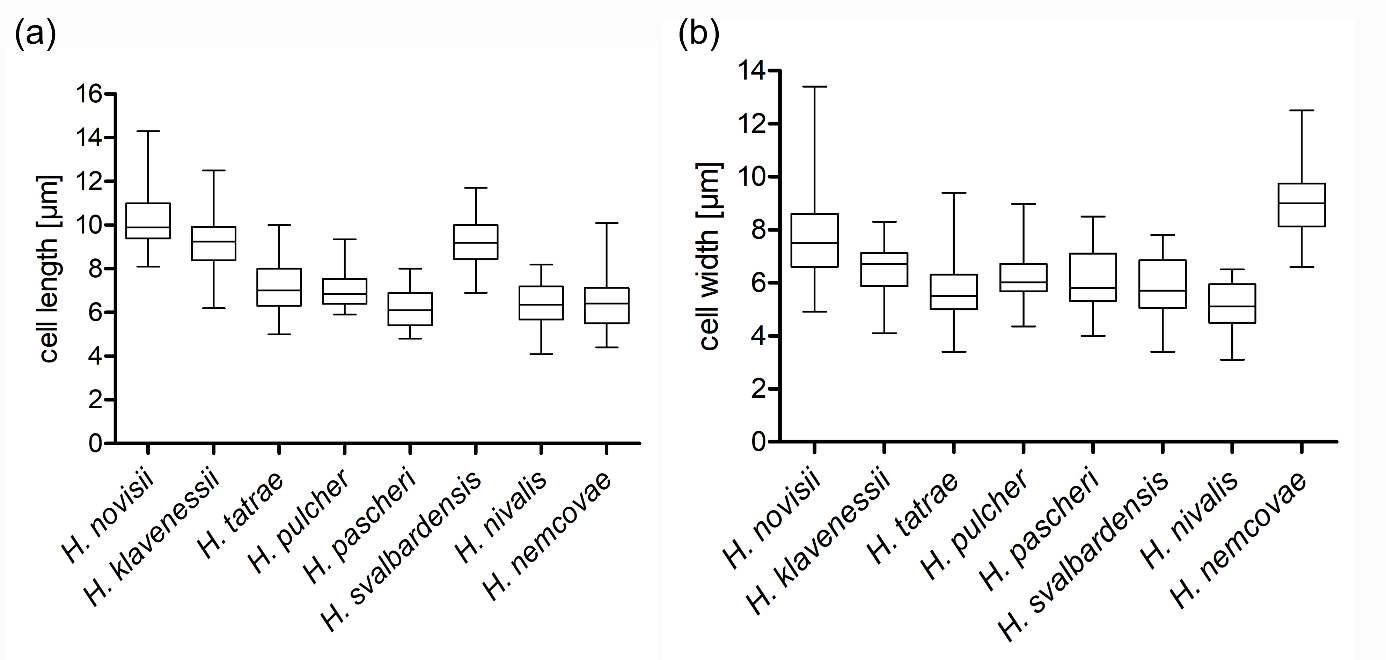


**Figure S2**. Cell widths and cell lengths of vegetative cells of eight new species of *Hydrurus* isolated from snow (n=30 for each strain). Note: the boxplot is drawn from quartile 1 to quartile 3 with a horizontal line drawn in the middle to denote the median. Whiskers (vertical lines) indicate actual minimum and maximum values in the dataset.
