# Supplementary material for: Novel Hydrurus species (Chrysophyceae) and their adaptations to high‐altitude European and Arctic snowfields
Source: J Phycol. 2026 Apr 29;62(3):818–45. doi: 10.1111/jpy.70162 (PMC13280783; doi:10.1111/jpy.70162)
Supplement: Supplementary file 3 — Figure S3. Field view of the type localities (highlighted by shovel or/and with arrow) of the eight new Hydrurus snow dwelling species described based on newly established algal strains in the course of this study: (a–m) Central Europe, (n–p) high Arctic. In detail, (a) H. pulcher CCCryo533a‐19, (b, c) H. tatrae WP195, (d, e) H. klavenessii WP222.2, (f) H. nivalis WP225, (g, h) H. pascheri WP227 (i, j), H. novisii WP264, (k–m) H. nemcovae WP271, (n–p) H. svalbardensis WP301. Order of the strains corresponds to the date of cryoflora sampling. Habitat description of localities including geographical data are shown in Table 1. [file JPY-62-818-s007.docx]

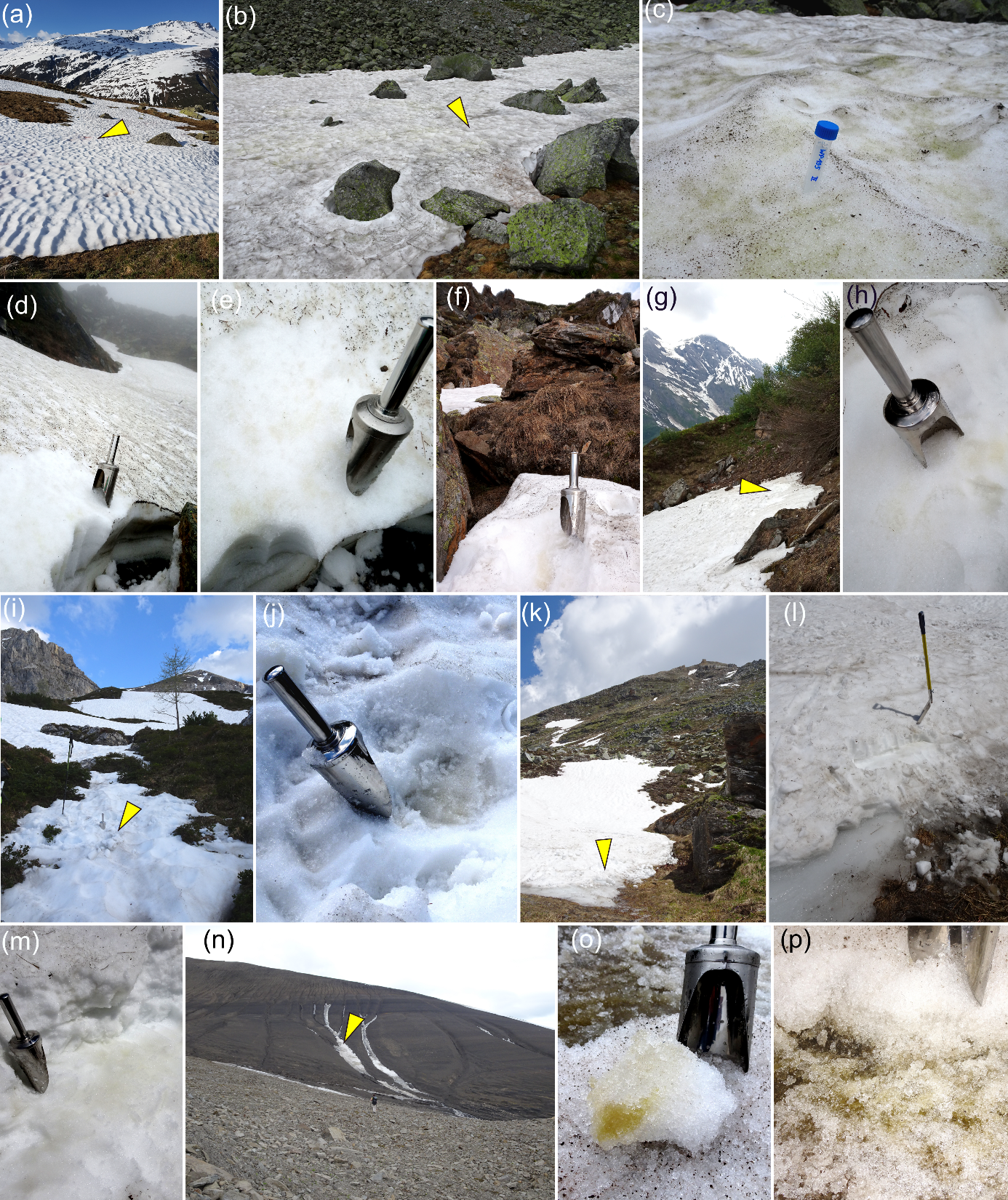


**Figure S3.** Field view of the type localities (highlighted by shovel or/and with arrow) of the eight new *Hydrurus* (*H*.) snow dwelling species described based on newly established algal strains in course of this study: (a-m) Central Europe, (n-p) high Arctic. In detail, (a) *H. pulcher* CCCryo533a-19, (b, c) *H. tatrae* WP195, (d, e) *H. klavenessii* WP222.2, (f) *H. nivalis* WP225, (g, h) *H. pascheri* WP227 (i, j), *H. novisii* WP264, (k-m) *H. nemcovae* WP271, (n-p) *H. svalbardensis* WP301 (with the first author as a scale bar). Order of the strains correspond to the date of cryoflora sampling. Habitat description of localities including geographical data are shown in **Table 1**.
