# Supplementary material for: Novel Hydrurus species (Chrysophyceae) and their adaptations to high‐altitude European and Arctic snowfields
Source: J Phycol. 2026 Apr 29;62(3):818–45. doi: 10.1111/jpy.70162 (PMC13280783; doi:10.1111/jpy.70162)
Supplement: Supplementary file 4 — Figure S4. Bayesian phylogenetic tree based on the rbcL gene. The newly obtained sequences from snow algal strains are in blue bold. Accession numbers, strain and field sample codes are indicated after each species name. The scale bar shows the estimated number of substitutions per site. Posterior probabilities (0.95 or more) and bootstrap values from maximum likelihood analyses (50% or more) are shown. Full statistical support (1.00/100) is marked with an asterisk. Thick branches represent nodes receiving the highest posterior probability support (1.00). [file JPY-62-818-s006.docx]

**
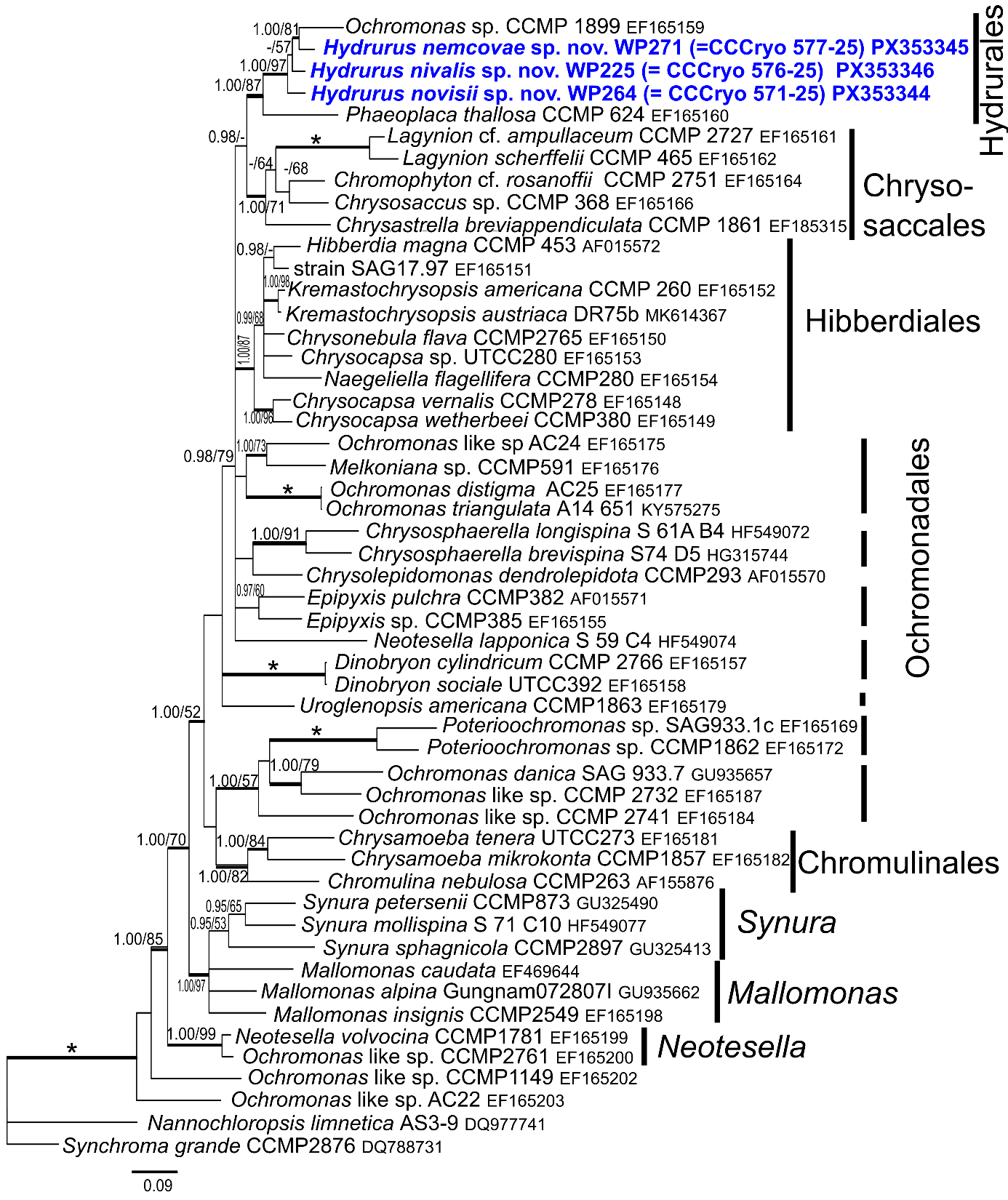
Figure S4.** Bayesian phylogenetic tree based on the *rbc*L gene. The newly obtained sequences from snow algal strains are in bold. Accession numbers, strain and field sample codes are indicated after each species name. The scale bar shows the estimated number of substitutions per site. Posterior probabilities (0.95 or more) and bootstrap values from maximum likelihood analyses (50% or more) are shown. Full statistical support (1.00/100) is marked with an asterisk. Thick branches represent nodes receiving the highest posterior probability support (1.00).
