# Supplementary material for: Novel Hydrurus species (Chrysophyceae) and their adaptations to high‐altitude European and Arctic snowfields
Source: J Phycol. 2026 Apr 29;62(3):818–45. doi: 10.1111/jpy.70162 (PMC13280783; doi:10.1111/jpy.70162)
Supplement: Supplementary file 5 — Table S1. Recipe for Chrysophycea medium (DY‐V). [file JPY-62-818-s005.docx]

|  | | | | |  |
| --- | --- | --- | --- | --- | --- |
| **Table S1.**  Recipe for Chrysophycea medium (DY-V)  Modified version of Provasoli-Guillard NCMA- National Center for Marine Algae and Microbiota Culturing diversity | | | | |  |
| To prepare, begin with 950 mL of dH_2_O and dissolve the MES (2-(N-morpholino)ethanesulfonic acid) buffer; subsequently, add the components 1-9 and bring the final volume to 1 liter using dH_2_O. Adjust the pH to 6.2 with KOH. Avoid HCl. Autoclave. After autoclaving, add the Vitamins stock solution at sterile conditions (filtered). | | | | | |
| ***Stock n°*** | ***Component*** | ***Stock solution concentration [g ⋅ L^-1^ dH_2_O]*** | ***Quantity*** | ***Concentration in Medium [M]*** |  |
|  | MES | --- | 200 mg | 1.02 x 10^-3^ |  |
| 1 | MgSO_4_ 7H2O | 50 | 1 mL | 2.03 x 10^-4^ |  |
| 2 | KCl | 3 | 1 mL | 4.02 x 10^-5^ |  |
| 3 | NH_4_Cl | 2,68 | 1 mL | 5.01 x 10^-5^ |  |
| 4 | NaNO_3_ | 20 | 1 mL | 2.35 x 10^-4^ |  |
| 5 | NaH_2_PO_4_ H_2_O | 5 | 1 mL | 3.62 x 10^-5^ |  |
| 6 | H_3_BO_3_ | 0.8 | 1 mL | 1.29 x 10^-5^ |  |
| 7 | Na_2_SiO_3_ 9H_2_O | 14 | 1 mL | 4.93 x 10^-5^ |  |
| 8 | CaCl_2_ 2H_2_O | 75 | 1 mL | 6.76 x 10^-4^ |  |
| 9 | Trace Element solution | (see recipe) | 1 mL | --- |  |
| 10 | Vitamin solution | (see recipe) | 0.5 mL | --- |  |
|  |  |  |  |  |  |
| **Trace Element Solution** | | | | |  |
| To prepare the Trace Element Solution (stock 9), begin with 900 mL of dH_2_O and dissolve the EDTA. Next, dissolve each compound and bring the final volume to 1 liter. Autoclave. If precipitation occurs during storage, it can usually be re-dissolved by heating or by adding a small amount of sodium hydroxide to make the solution slightly more basic. | | | | | |
|  |  |  |  |  |  |
|  | ***Component*** | ***Stock Solution concentration [g ⋅ L^-1^ dH_2_O]*** | ***Quantity*** | ***Concentration in Medium [M]*** |  |
|  | Na_2_EDTA 2H_2_O | --- | 8.0 g | 2.15 x 10^-5^ |  |
|  | FeCl_3_ 6 H_2_O | --- | 1.0 g | 3.70 x 10^-6^ |  |
|  | MnCl_2_ 4 H_2_O | --- | 200 mg | 1.01 x 10^-6^ |  |
|  | ZnSO_4_ 7 H_2_O | --- | 40 mg | 1.39 x 10^-7^ |  |
| 1. | CoCl_2_ 6 H_2_O | 8 | 1 mL | 3.36 x 10^-8^ |  |
| 2. | Na_2_MoO_4_  2H_2_O | 20 | 1 mL | 8.27 x 10^-8^ |  |
| 3. | Na_3_VO_4_ 10H_2_O | 2 | 1 mL | 5.49 x 10^-9^ |  |
| 4. | H_2_SeO_3_ | 4 | 1 mL | 2.31 x 10^-8^ |  |
| To prepare the Vitamin Solution (10), begin with 950 mL of dH_2_O, dissolve the above components and bring the final volume to 1 L with dH_2_O. Adjust the pH to 7 and store in the fridge. | | | | | |
| Prepare solidified DY-V for petri dishes with 1.4 % agar (w/v)! Set pH after adding agar but prior autoclaving. | | | | |  |
| **Vitamin Solution** | | | | |  |
|  | **Component** | **Stock Solution Concentration [mg ⋅ L^-1^ dH_2_O]** |  |  |  |
|  | Vitamin B12 | 0.20 |  |  |  |
|  | Biotin | 1.0 |  |  |  |
|  | Thiamine-HCl (B1) | 100 |  |  |  |
|  | Niacinamide (B3) | 0.10 |  |  |  |
