# Supplementary material for: Novel Hydrurus species (Chrysophyceae) and their adaptations to high‐altitude European and Arctic snowfields
Source: J Phycol. 2026 Apr 29;62(3):818–45. doi: 10.1111/jpy.70162 (PMC13280783; doi:10.1111/jpy.70162)
Supplement: Supplementary file 6 — Table S2. Comparison of vegetative cells of six species of chrysophytes isolated so far from melting snow (C., Chromulina; K., Kremastochrysopsis; O., Ochromonas). [file JPY-62-818-s004.docx]

| **Table S2.** Comparison of vegetative cells of the six species of chrysophytes isolated from melting snow (*C. – Chromulina*, *K.- Kremastochrysopsis*, *O. - Ochromonas*) | | | | | | |
| --- | --- | --- | --- | --- | --- | --- |
|  | *C. chionophilia* | *C. ettlii* | *C.* cf. *elegans* | *K. austriaca* | *O. smithii* | *O. itoi* |
| flagellate form | ovoid, flattened in cross section | spherical | spherical to ellipsoidal, rarely pointed posterior cell end | spherical to pyriform to oval | tetrahedral  (+ gametes) | liver-shaped, triangular, nearly rhombic or nearly pear-shaped |
| eyespot | tiny | ellipsoidal or linear | absent | absent | absent | present |
| cell width | 5-6 µm | 7-9 µm | 4.5-6 µm | 5-8 µm | 15-22 µm | 3-4 µm |
| cell length | 5-12 µm | 7-9 µm | 4.5-7 µm | 5-8 µm | 12-21 µm | 3.5-5 µm |
| stomatocysts | unknown | unknown | unknown | unknown | no details | unknown |
| chloroplasts | parietal, cup-shaped or laminate | parietal, cup-shaped | parietal, cup-shaped | parietal | 2, bord shaped | 2, disc shaped |
| number of flagella (LM) | 1 | 1 | 1(2) | 1 | 2(1) | 1(2) |
| length of flagellum | that of the cells | almost twice as long as the cell | that of the cells | almost three times as long as the cell | a little shorter than body length | that of the cells |
| reference | Stein (1963),  Hoham et Blinn (1979),  Sutton (1970) | Hindák (1969) | Novis (2002),  Lukavsky et al. (2009) | Remias et al. (2020) | Fukushima (1963) | Fukushima (1963) |

**References**

Fukushima, H. (1963). Studies on cryophytes in Japan*. Journal of the Yokohama Municipal University*, *series C*, *43*(144), 1–146.

Hindák, F. (1969). Brownish snow in the High Tatras. *Biológia* (Bratislava), *24*, 80–85.

Hoham, R. W., & Blinn, D. W. (1979). Distribution of cryophilic algae in an arid region, the American Southwest. *Phycologia*, *18*, 133–145.

Lukavský, J., Furnadzhieva, S., & Nedbalová, L. (2009). First record of cryoseston in the Vitosha Mountains (Bulgaria). *Nova Hedwigia*, *88* (1–2), 97–109.

Novis, P. M. (2002). New records of snow algae for New Zealand, from Mt Philistine, Arthur's Pass National Park. *New Zealand Journal of Botany*, *40*(2), 297–312.

Remias, D., Procházková, L., Nedbalová, L., & Andersen, R. A. (2020). Two new *Kremastochrysopsis* species, *K. austriaca* sp. nov. and *K. americana* sp. nov. (Chrysophyceae). *Journal of Phycology*, *56*(1), 135–145.

Stein, J. R. (1963). A *Chromulina* (Chrysophyceae) from snow*. Canadian Journal of Botany*, 41(9): 1367–1370.

Sutton, E. A. (1970). *The physiology and life histories of selected cryophytes of the Pacific Northwest* [Doctoral dissertation, Oregon State University].
