# Supplementary material for: Novel Hydrurus species (Chrysophyceae) and their adaptations to high‐altitude European and Arctic snowfields
Source: J Phycol. 2026 Apr 29;62(3):818–45. doi: 10.1111/jpy.70162 (PMC13280783; doi:10.1111/jpy.70162)
Supplement: Supplementary file 7 — Table S3. Comparison of vegetative cells of the eight species of Hydrurus isolated from melting snow. [file JPY-62-818-s001.docx]

| **Table S3.** Comparison of vegetative cells of the eight species of *Hydrurus* (*H*.) isolated from melting snow. | | | | | | | | |
| --- | --- | --- | --- | --- | --- | --- | --- | --- |
|  | *H. novisii* | *H. klavenessii* | *H. tatrae* | *H. pulcher* | *H. pascheri* | *H. svalbardensis* | *H. nivalis* | *H. nemcovae* |
| flagellate form | broadly ovoid | ovoid | ovoid; mainly capsal | ovoid | ovoid | elongated, dorsoventrally flattened | ovoid, dorsoventrally flattened | ovoid, dorsoventrally flattened |
| eyespot | absent | absent | absent | absent | absent | linear | ellipsoid | ellipsoid |
| cell width | 4.9-13.4 µm | 4.1-8.3 µm | 3.4-9.4 µm | 4.4-9.0 µm | 4.0-7.8 µm | 3.4-7.8 µm | 3.1-6.5 µm | 4.4-10.1 µm |
| cell length | 8.1-14.3 µm | 6.2-12.5 µm | 5.0-9.8 µm | 5.9-9.4 µm | 4.8-8.5 µm | 6.9-11.7 µm | 4.1-8.2 µm | 6.6-13.7 µm |
| stomatocysts | unknown | unknown | unknown | unknown | unknown | unknown | unknown | unknown |
| chloroplasts | parietal | 1-2, parietal | parietal | 1-2, parietal | parietal | parietal | parietal | parietal |
| flagella (LM) | 1 | 1 | 1 | 1 | 1 | 1 | 1(2) | 1 |
| short flagella length (SEM) | 0.5 µm | 3 µm | n.d. | 0.5 µm | 0.5 µm | 1 µm | 1 µm | 0.5 µm |
| length of flagellum | almost 3x as long as the cell | almost 3x as long as the cell | n.d. | up to 1.5x as cell | that of the cells | almost 2x as long as the cell | almost 2x as long as the cell | almost 2x as long as the cell |
| tetrahedral flagellate | present | present | present (rarely) | present (rarely) | present | absent | absent | absent |
| reference | this study | this study | this study | this study | this study | this study | this study | this study |
