# Supplementary material for: Novel Hydrurus species (Chrysophyceae) and their adaptations to high‐altitude European and Arctic snowfields
Source: J Phycol. 2026 Apr 29;62(3):818–45. doi: 10.1111/jpy.70162 (PMC13280783; doi:10.1111/jpy.70162)
Supplement: Supplementary file 8 — Table S4. Cellular fatty acid composition of Hydrurus (H.) sp. field samples (f) (WP203, WP395, and WP401), Hydrurus svalbardensis field (f) and strain (s) samples (WP301) in [%] of total fatty acids (TL; all samples) and in [%] of the three major lipid classes: Neutral lipids (NL), phospholipids (PL), and glycolipids (GL). The table shows only fatty acids that had abundances greater than 0.1%, “*” bacterial contamination. The relative proportion of saturated (SAFA), monounsaturated (MUFA), and polyunsaturated (PUFA) fatty acids is also given. Values of WP301 strain are means of three independent biological replicates (±SD). [file JPY-62-818-s002.docx]

|  | | ***H.* sp.** | | | | | ***H.* sp.** | | ***H.* sp.** | | ***H. svalbardensis*** | |
| --- | --- | --- | --- | --- | --- | --- | --- | --- | --- | --- | --- | --- |
| **Fatty acids** | | **WP203f** | | | | | **WP395f** | | **WP401f** | | **WP301s** | **WP301f** |
| **FA** | **TL** | | | **NL** | **PL** | **GL** | **TL** | **TL** | | **TL** | | **TL** |
| 14:0 | | | 7.7 | 12.9 | 0.0 | 6.0 | 18.3 | 20.2 | | 8.4±0.5 | | 9.0 |
| 15:0* | | | 0.1 | 0.2 | 0.1 | 0.0 | 0.0 | 0.0 | | 3.1± 0.4 | | 1.9 |
| 16:0 | | | 16.7 | 18.5 | 4.2 | 31.8 | 11.1 | 16.5 | | 5.8±0.2 | | 7.5 |
| i16:0* | | | 0.2 | 0.3 | 0.3 | 0.0 | 0.0 | 0.0 | | 0.0 | | 0.0 |
| 16:1 (9Z) | | | 0.3 | 0.1 | 0.7 | 0.0 | 4.6 | 0.0 | | 1.6±0.2 | | 1.7 |
| 16:2 (7Z, 10Z) | | | 0.0 | 0.0 | 0.0 | 0.0 | 0.3 | 0.5 | | 0.0 | | 0.0 |
| 16:3 (7Z, 107, 13Z) | | | 0.2 | 0.3 | 0.0 | 0.0 | 0.3 | 0.0 | | 0.0 | | 0.0 |
| 16:4 (4Z,7Z,19Z,13Z) | | | 0.2 | 0.4 | 0.0 | 0.0 | 0.2 | 0.3 | | 0.0 | | 0.0 |
| 17:0* | | | 0.1 | 0.1 | 0.0 | 0.1 | 0.0 | 0.0 | | 5.0±0.2 | | 5.6 |
| 18:0 | | | 19.0 | 15.2 | 11.2 | 41.5 | 5.4 | 7.1 | | 5.2±0.5 | | 4.9 |
| 18:1 (9Z) | | | 21.5 | 33.8 | 10.7 | 5.6 | 25.4 | 22.3 | | 12.6±0.7 | | 15.1 |
| 18:1 (11Z) | | | 0.8 | 0.4 | 1.8 | 0.1 | 1.1 | 0.5 | | 1.2±0.2 | | 1.1 |
| 18:2 (9Z, 12Z) | | | 9.3 | 3.2 | 25.3 | 0.5 | 5.7 | 9.4 | | 9.5±0.4 | | 7.4 |
| 18:3 (6Z, 9Z, 12Z) | | | 1.1 | 0.0 | 3.7 | 0.0 | 0.0 | 0.0 | | 1.1±0.2 | | 1.1 |
| 18:3 (9Z, 12Z, 15Z) | | | 4.0 | 1.4 | 6.6 | 7.0 | 3.5 | 3.1 | | 5.6±0.6 | | 5.8 |
| 18:4 (6Z, 9Z, 12Z, 15Z) | | | 18.5 | 12.9 | 35.3 | 7.1 | 8.7 | 6.4 | | 25.7±0.8 | | 22.2 |
| 20:2 (11Z, 14Z) | | | 0.0 | 0.0 | 0.0 | 0.0 | 3.7 | 2.7 | | 0.3±0.2 | | 0.0 |
| 20:3 (8Z, 11Z, 14Z) | | | 0.0 | 0.0 | 0.0 | 0.0 | 0.4 | 0.7 | | 2.2±0.1 | | 4.2 |
| 20:3 (11Z, 14Z, 17Z) | | | 0.0 | 0.0 | 0.0 | 0.0 | 0.2 | 0.2 | | 0.0 | | 0.0 |
| 20:4 (5Z, 8Z, 11Z, 14Z) | | | 0.0 | 0.0 | 0.0 | 0.0 | 1.5 | 0.9 | | 0.3±0.1 | | 0.9 |
| 20:5 (5Z, 8Z, 11Z, 14Z, 17Z) | | | 0.0 | 0.0 | 0.0 | 0.0 | 2.0 | 0.9 | | 3.7±0.2 | | 3.4 |
| 22:5 (7Z, 10Z, 13Z, 16Z, 19Z) | | | 0.0 | 0.0 | 0.0 | 0.0 | 4.2 | 2.5 | | 0,3±0.1 | | 0.9 |
| 22:6 (4Z, 7Z, 10Z, 13Z, 16Z, 19Z) | | | 0.0 | 0.0 | 0.0 | 0.0 | 3.3 | 6.1 | | 8.2±0.4 | | 7.1 |
| SAFA | | | 43.9 | 47.5 | 15.9 | 79.7 | 34.8 | 43.7 | | 27.5 | | 29.0 |
| MUFA | | | 22.7 | 34.3 | 13.2 | 5.7 | 31.2 | 22.8 | | 15.5 | | 18.0 |
| PUFA | | | 33.4 | 18.2 | 70.9 | 14.6 | 34.0 | 33.5 | | 57.0 | | 53.0 |

**Table S4.** Cellular fatty acid composition of *Hydrurus* (*H.*) sp. field samples (*f*), *Hydrurus svalbardensis* field (*f*) and strain (*s*) samples in [%] of total fatty acids (TL; all samples) and in [%] of the three major lipid classes (WP203): Neutral lipids (NL), phospholipids (PL), and glycolipids (GL). The table shows only fatty acids that had abundances greater than 0.1%, “*” - bacterial contamination. The relative proportion of saturated (SAFA), monounsaturated (MUFA), and polyunsaturated (PUFA) fatty acids is also given. Values of WP301 strain are means of three independent biological replicates (±SD).
